# Versatile microbial community responsible for nitrate turnover in a carbonate aquifer in southwest Germany

**DOI:** 10.1093/femsec/fiag065

**Published:** 2026-06-30

**Authors:** Sergey Abramov, Nia Blackwell, Karsten Osenbrück, Daniel Straub, Sven Nahnsen, Andreas Kappler, Peter Grathwohl, Sara Kleindienst

**Affiliations:** Department of Geoscience, University of Tuebingen, 72076 Tuebingen, Germany; Institute of Sanitary Engineering, Water Quality and Solid Waste Management, University of Stuttgart, 70569 Stuttgart, Germany; Department of Geoscience, University of Tuebingen, 72076 Tuebingen, Germany; Department of Geoscience, University of Tuebingen, 72076 Tuebingen, Germany; Federal Institute for Geosciences and Natural Resources (BGR), 30655 Hannover, Germany; Department of Geoscience, University of Tuebingen, 72076 Tuebingen, Germany; Quantitative Biology Center (QBiC), University of Tuebingen, 72076 Tuebingen, Germany; Quantitative Biology Center (QBiC), University of Tuebingen, 72076 Tuebingen, Germany; Institute for Bioinformatics and Medical Informatics, University of Tuebingen, 72076 Tuebingen, Germany; Department of Geoscience, University of Tuebingen, 72076 Tuebingen, Germany; Cluster of Excellence: EXC 2124, Controlling Microbes to Fight Infection, University of Tuebingen, 72076 Tuebingen, Germany; Cluster of Excellence: EXC 3121, TERRA – Terrestrial Geo-Biosphere Interactions in a Changing World, University of Tuebingen, 72076 Tuebingen, Germany; Department of Geoscience, University of Tuebingen, 72076 Tuebingen, Germany; Department of Geoscience, University of Tuebingen, 72076 Tuebingen, Germany; Institute of Sanitary Engineering, Water Quality and Solid Waste Management, University of Stuttgart, 70569 Stuttgart, Germany

**Keywords:** groundwater, karst, carbonate aquifer, denitrification, subsurface microorganisms

## Abstract

Denitrifiers contribute to the remediation of agriculturally impacted aquifers by using NO_3_⁻ as an electron acceptor. However, the effects of local hydrogeochemical factors (e.g. O_2_, electron donors, carbon sources) on the abundance of denitrifiers remain poorly understood. To address this, we sampled planktonic (0.2–0.4 µm, 0.4–8.0 µm) and particle-associated (>8 µm) biomass from nine groundwater wells and one karstic spring in the Ammer River catchment (SW Germany). Comparing groundwater hydrochemistry with microbial community composition and the relative abundance of 16S rRNA and N-cycling genes (*nirK, nirS, amoA*) revealed a correlation between declining O_2_ and dissolved organic carbon levels in the aquifer and lower bacterial and archaeal 16S rRNA gene copy numbers. Despite oxic conditions in the recharge zone, NO_3_⁻ could be reduced heterotrophically and autotrophically in anoxic microniches. In the confined anoxic zone, taxa such as *Aquabacterium, Acidovorax*, and *Gallionella* could couple NO_3_⁻ reduction to pyrite-derived Fe(II) oxidation. Microorganisms such as *Rhodoferax, Sediminibacterium*, and *Sulfurifustis* could oxidize pyrite-derived reduced sulfur compounds. Hydrogen-oxidizing (e.g. *Hydrogenophaga*) and CH_4_-oxidizing microorganisms (e.g. *Candidatus* Methylomirabilis) could also contribute to NO_3_⁻ turnover. These findings suggest that the turnover of NO_3_^-^ in the carbonate aquifer shifts from pathways predominantly supported by organic matter in recharge groundwater towards an increasing reliance on pyrite-derived electron donors under anoxic conditions.

## Teaser

Graphical abstract showing that heterotrophic and lithotrophic denitrifying microorganisms contribute to the turnover of nitrate in the carbonate aquifer of the Ammer River in south-west Germany.

## Introduction

Karstified and fractured aquifers represent an important source of freshwater worldwide (Chen et al. 2017). Due to their distinctive hydrogeological structure, these aquifers often respond rapidly to hydrometeorological events, which can strongly influence groundwater flow, solute transport and water quality (Hartmann et al. 2014). As a result, they are particularly vulnerable to contamination from agricultural activities.

The application of NH_4_⁺-based fertilizers may lead to the leaching of reactive nitrogen compounds into groundwater. If not assimilated by plants, NH_4_⁺ may enter the soil nitrogen cycle, being converted to NO_3_⁻ through nitrification or comammox (Bijay and Craswell 2021). Because of its high solubility and mobility in subsurface environments, NO_3_⁻ is considered one of the most widespread groundwater contaminants worldwide (Abascal et al. 2022). In Germany, groundwater NO_3_⁻ concentrations are among the highest reported within the European Union (European Commission 2018). The Ammer Valley (SW Germany) contains a karstified and fractured carbonate aquifer that is used for producing drinking water and exhibits a pronounced NO_3_⁻ gradient. Nitrate concentrations decrease from ∼60 mg/l in the unconfined (recharge) zone to ∼1 mg/l in the confined (anoxic) zone (Utom et al. 2020). This gradient suggests intensive NO_3_⁻ turnover along groundwater flow paths (Utom et al. 2020, Visser et al. 2021). However, it remains largely unknown which microorganisms are involved in denitrification and, ultimately, which electron donors may promote the reduction of NO_3_⁻.

Previous studies have demonstrated the presence of autotrophic, NO_3_⁻-reducing, Fe(II)-oxidizing (NRFeOx) microorganisms in the Ammer aquifer through the enrichment of cultures from groundwater samples (Jakus et al. 2021a,b, Huang et al. 2022). These findings suggest that microorganisms capable of coupling NO_3_⁻ reduction to Fe(II) oxidation are present in the aquifer and may contribute to autotrophic denitrification under anoxic conditions. The Fe(II) supporting this process is likely supplied by Fe(II)-bearing minerals, including pyrite, present within the aquifer matrix. However, NRFeOx microorganisms likely represent only one component of a broader microbial community involved in NO_3_⁻ turnover. In addition, NO_3_⁻ reduction can be driven by microorganisms utilizing other electron donors, including dissolved organic carbon (DOC) (Petrova et al. 2022). To understand how groundwater chemistry shapes microbial community composition and NO_3_⁻ turnover in the Ammer aquifer, we analyzed planktonic and particle-associated microbial communities along a hydrochemical gradient. Specifically, we sampled groundwater from monitoring wells and production wells, as well as from a karstic spring, and determined the composition of microbial communities. Therefore, the objectives of this study were i) to determine the influence of hydrogeochemical parameters (e.g. DOC, O_2_, NO_3_⁻ concentrations) on denitrifying microbial communities, and ii) to identify microorganisms associated with NO_3_⁻ turnover.

## Methods

### Study area

**Figure 1:**
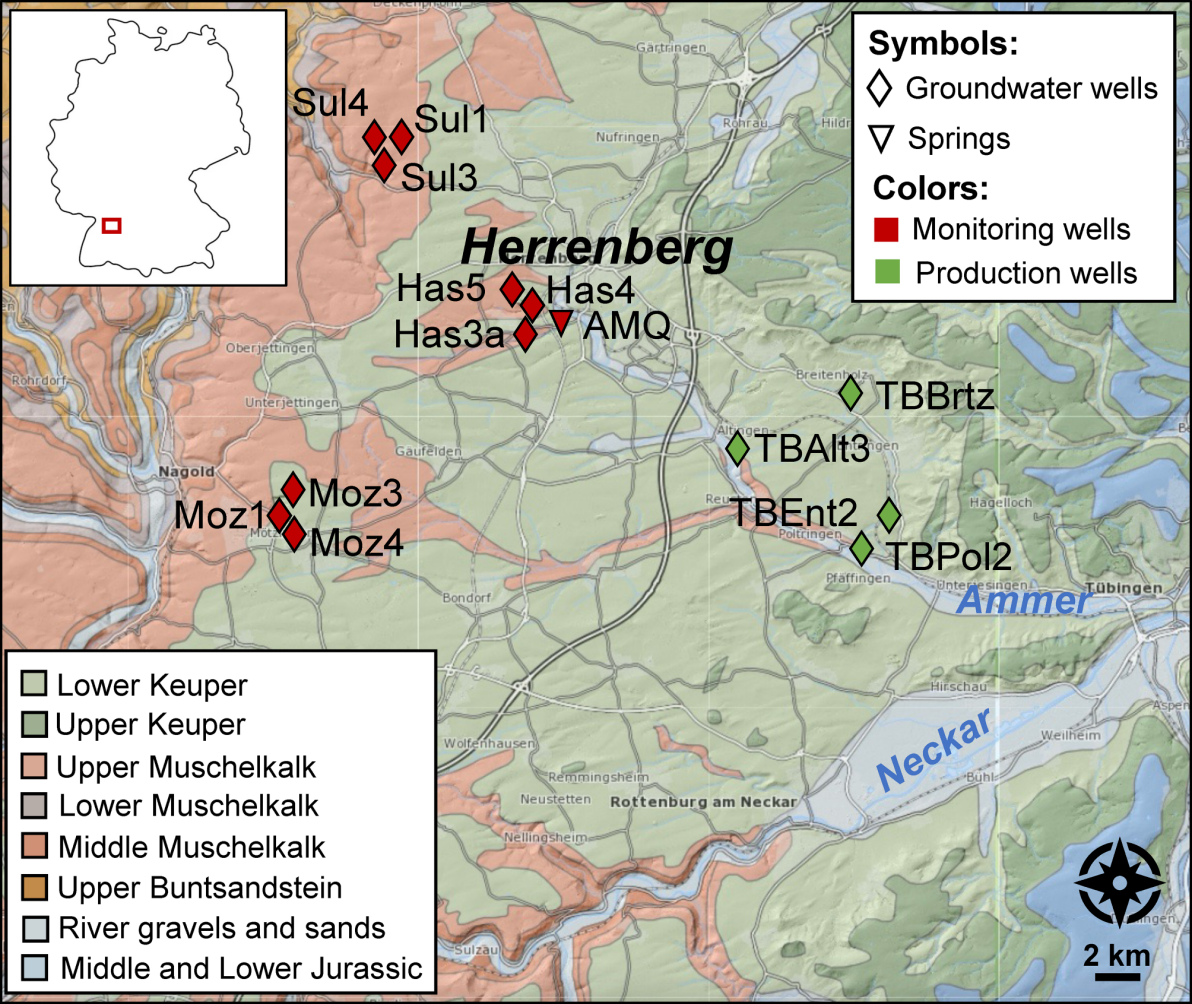
Location and geology of the sampling sites (monitoring wells, production wells and karstic spring) at the Ammer River catchment (SW Germany). Generated with maps.lgrb-bw.de.

The “Oberes Gäu” is an important agricultural region in Baden-Württemberg, SW Germany. Here, the study area is located between the residential areas of Mötzingen, Sulz am Eck, Ammerbuch, Herrenberg, Entringen, and Poltringen (Fig. 1; Table S1) in the catchment of the Ammer River, a tributary of the Neckar River. The field sites included the monitoring wells Sul1, Sul4, Sul3, Moz1, Moz3, Moz4, Has3a, Has4, Has5, and the karstic Ammer spring (AMQ) located in the recharge area, as well as the production wells TBAlt3, TBEnt2, TBPol2, and TBBrtz located in the anoxic zone of the aquifer. All field sites were located at altitudes ranging from 576 m asl to 350 m asl (Table S1).

### Geological and hydrogeological setting

The study area is dominated by Triassic rock formations of the Upper Muschelkalk and the Lower and Middle Keuper (Fig. 1). The Upper Muschelkalk rock formations constitute a regional fractured aquifer spanning ∼150 km^2^ (D’Affonseca et al. 2020).

At the sites Sul4, Has4, Moz3, Moz4, Has5, Has4, and Has3a, the aquifer is covered by thin Quaternary deposits. At the sites TBAlt3, TBPol2, and TBEnt2, the Upper Muschelkalk aquifer is overlain by low-permeable Upper Triassic rocks that increase in thickness towards the SE. The Upper Muschelkalk consists of fractured and partly karstified limestones and dolomites with a total thickness of 80-90 m (Villinger 1982). The base of the aquifer is formed by porous to cavernous dolomites and dolomitic marls, overlying clayey subrosional residues of the evaporite-dominated Middle Muschelkalk formations. Much of the Upper Muschelkalk consists of a series of low-porosity (0.5%–5.0%) micritic limestones. Typical features of the Muschelkalk formation are Fe(II)-bearing minerals (e.g. pyrite and siderite). Within the limestone matrix, small pyrite crystals (FeS_2_) can be frequently found with concentrations of up to 2 mass-% (Visser et al. 2021, Osenbrück et al. 2022). The organic carbon content of the limestones is generally low, with <0.06 mass-%. The upper 15–20 m of the Upper Muschelkalk consists of porous (15%–30%) or partly cavernous dolomites and dolomitic limestones interbedded with thin marl beds of constant thickness (Trigonodus Dolomite) (Osenbrück et al. 2022). The groundwater flow direction is from NW to SE, following the dipping of the Triassic formations.

### Sampling

In January and February 2018, groundwater samples were collected for hydrochemical and molecular biological analyses from nine monitoring wells, four water supply wells, and one karstic spring, which together form a hydrologically connected system. All of these wells extract groundwater from the Upper Muschelkalk aquifer (Table S1). The main difference between monitoring and production wells is that supply wells are equipped with pumps and operate at higher pumping rates. They are also typically run continuously, thereby integrating groundwater over larger capture zones. In contrast, monitoring wells are sampled at low flow rates, representing more localized conditions. Due to logistical constraints associated with processing a large number of sites, including the need for laboratory-based filtration and thorough cleaning and sterilization of sampling equipment between sites, sampling was conducted sequentially rather than in parallel.

Submersible pumps, which were cleaned and sterilized with ethanol (80%) in the field, were used to sample the monitoring wells. Groundwater samples were taken after the volume of water in the wells had been replaced at least three times. Pumps were typically operated at flow rates between 0.01 and 0.16 l/s. Water samples at the water supply wells were taken from a sterilized tap at the wellhead. At the Ammer spring, samples were taken as grab samples directly at the outlet. At each site, the groundwater was poured directly into two sterile 10 l Nalgene carboys.

Dissolved oxygen (DO), conductivity (EC), temperature, and pH were measured in the field using hand-held probes (WTW GmbH). Additional samples for major and minor anions were collected in the field, immediately filtered to <0.45 µm and stored at 4°C until analysis. Samples for Fe analysis were filtered to <0.45 µm in the field and fixed in 1 M HCl:40 mM sulfamic acid (1 : 1).

Groundwater for gas analysis was sampled by pumping it into a 10 l container via a tube placed at the bottom, to avoid bubble formation. After allowing for several minutes of overflow, 50 ml headspace vials were filled underwater and sealed to prevent air contact. The samples were transported to the lab on the same day, where a N_2_ headspace was introduced. Following equilibration, the H_2_, N_2_O, and CH_4_ concentrations in the headspace were determined using gas chromatography. Dissolved H₂ concentrations were calculated using Henry’s law.

Samples for molecular biological analyses were taken only from the Sul4, Sul3, Moz1, Has4, Has5, AMQ, TBAlt3, TBEnt2, TBPol2, and TBBrtz. The samples were then transported immediately back to the laboratory, where they were filtered upon arrival. Samples were filtered sequentially through sterile polycarbonate filters with pore sizes of 8 µm, 0.4 µm, and 0.2 µm (Merck Millipore). This sequential filtration approach was applied to separate particle-associated communities (>8 µm) from predominantly free-living microbial communities (0.45–0.2 µm). Due to the high particle loads in the groundwater samples, sequential filtration was necessary to prevent the rapid clogging of fine-pore filters. This approach will not fully exclude overlap between size fractions, as smaller cells may be retained on larger pore-size filters due to clogging or attachment to particles during filtration. Sessile microbial communities directly attached to aquifer rock surfaces were not specifically sampled in this study. However, the >8 µm particle-associated fraction may have included detached biofilm material. The filters were stored at −80°C until further processing.

### Hydrogeochemical analysis

Nitrate concentrations were determined in <0.45 µm filtered samples by spectrophotometric analysis using a continuous flow analyser (CFA, Seal Analytics, AA3). Major dissolved ions were quantified in the laboratory by ion chromatography (Thermo Scientific Dionex DX-120; Anions: Dionex IonPac A523 analytical column with a Dionex IonPac AG23 guard column; cations: Dionex Ion-Pac CS12A-5-μm analytical column with a Dionex IonPac CG12A-5-μm guard column; LOQ = 0.1 mg/l). Dissolved organic carbon was quantified on a highTOC II-analyser (Elementar) after acidification to pH 2 and rinsing to remove the inorganic carbon (LOQ = 0.8 mg/l).

Samples for Fe quantification were stored at 4°C and subsequently analyzed using the modified ferrozine assay (Klueglein and Kappler 2013), with Fe measured photometrically at 562 nm (Thermo Scientific Multiskan GO).

### DNA extraction and 16S rRNA gene sequencing

DNA was extracted from the filters using the FastDNA® Spin Kit (MP Biomedicals) according to the manufacturer’s instructions. No extraction blanks, field blanks or PCR-negative controls were included in this study. Standard contamination prevention procedures were applied throughout sampling and laboratory processing, including the use of sterile consumables, 80% ethanol-cleaned equipment and processing in a clean molecular laboratory environment. The DNA concentration of each sample was measured using a Qubit 2.0 Fluorimeter with DNA HS Kits and stored at −80°C until further analysis. Due to the low concentration of DNA in some samples, either the extracted DNA was used directly for 16S rRNA gene amplification or a preliminary booster PCR was performed, and an aliquot of the resulting amplicon was used for sequencing. Further details on the booster PCR procedure, including the number of additional amplification cycles per sample, are provided in the Supporting Information (Table S2). Two-step amplification strategies are commonly used for low-biomass samples and have been shown not to substantially alter microbial community composition compared to standard single-step amplification protocols (Breyer et al. 2024).

Briefly, for library preparation, DNA or an aliquot of the amplicon from the preliminary PCR were subjected to amplification with universal primers [515F: GTGYCAGCMGCC-GCGGTAA (Parada et al. 2016) and 806R: GGACTACNVGGGT-WTCTAAT (Apprill et al. 2015)] fused to Illumina adapters. These primers target the 16S rRNA gene and are designed to capture both Bacteria and Archaea. Each PCR reaction for sequencing contained: 12.5 µl of KAPA Hifi ReadyMix PCR Kit (KAPA BioSystems, Cape Town, South Africa), 0.5 µl of each of the tagged universal primers, 10.5 µl of sterile H_2_O, and 1 µl of extracted DNA or amplicon. The following PCR programs were used for library preparation: initial denaturation for 3 min at 95°C, between 20 and 25 cycles denaturation for 30 s at 95°C, annealing for 30 s at 55°C, and extension for 30 s at 72°C. These steps were followed by a final elongation step at 72°C for 5 min. The quality of amplicons was verified by agarose gel electrophoresis and NanoDrop (NanoDrop 1000, ThermoScientific, Waltham, MA, USA). Samples were sent for analysis and subsequent library preparation steps and Illumina MiSeq sequencing (Illumina, San Diego, CA, USA) using the 2 × 250 bp MiSeq Reagent Kit v2 (500 cycles kit) were performed at Microsynth AG (Balgach, Switzerland).

Between 14 137 and 237 771 read pairs were obtained for each of the samples (in total 7 739 825 read pairs) in two sequencing runs. Data processing, including quality control, reconstruction of sequences, and taxonomic annotation was done using nf-core/ampliseq v2.11.0 (Straub et al. 2020) of the nf-core collection of workflows (Ewels et al. 2020). The pipeline was executed with Nextflow v24.04.4 (Di Tommaso et al. 2017) and Singularity v3.8.7 (Kurtzer et al. 2017). Samples from each sequencing run were treated separately for preprocessing. Primers were trimmed and untrimmed sequences were discarded (<21% per sample) with Cutadapt version 4.6 (Martin 2011). Adapter and primer-free sequences were pooled per sequencing run with DADA2 v1.30.0 (Callahan et al. 2016) to eliminate PhiX contamination, trim reads (before median quality drops below 35; forward reads were trimmed at 230 bp and reverse reads at 229 bp), correct errors, merge read pairs, and remove polymerase chain reaction (PCR) chimeras. Ultimately, 41 461 amplicon sequencing variants (ASVs) were obtained across all samples and sequencing runs. 41 129 ASVs were between 240 bp and 270 bp in length, the remaining 332 ASVs, representing in average 0.31% read counts per sample (<3.78%), were deemed spurious and discarded. Taxonomic classification was performed with DADA2 and the SILVA v138.1 database (Quast et al. 2013). Intermediate results were imported into QIIME2 version 2023.7.0 (Bolyen et al. 2019). 5 030 ASVs were present in fewer than three samples or were classified as chloroplasts or mitochondria and were removed, totalling 0.05%–79.44% (average 3.61%) relative abundance per sample, and retaining 36 099 ASVs across all samples. The final abundance table had 10 185–167 860 read counts per sample (total 5 541 833 read counts). Alpha rarefaction curves were produced with the QIIME2 diversity alpha-rarefaction plugin, which indicated that the richness of the samples had been fully observed. The abundance table was rarefied by randomly subsampling to a sampling depth of 10 185 and Bray–Curtis dissimilarity (Sørensen 1948) as well as Shannon’s index (Shannon and Weaver 1949) were calculated with q2-diversity (https://github.com/qiime2/q2-diversity). Raw sequencing data has been deposited at NCBI in the Sequence Read Archive (SRA) under BioProject accession number PRJNA1250814 (https://www.ncbi.nlm.nih.gov/bioproject/PRJNA1250814). Relative abundances were not normalized for variation in the number of 16S rRNA gene copies or primer bias, which may influence the apparent abundance of some taxa, such as *Pseudomonas*. Therefore, relative abundances should be interpreted cautiously as they may not reflect absolute cell numbers.

### Quantitative PCR of target genes

Quantitative PCR on DNA extracts was performed in triplicate for each sample using the SsoAdvanced Universal SYBRGreen® Supermix (Bio-Rad Laboratories GmbH, Munich, Germany) on an iQ5 real-time PCR detection system (iQ5 optical system software, version 2, Bio-Rad). Triplicate measurements were calculated for each sample per run and data were analyzed using the iQ5 optical system software, version 2.0 (BioRad). The quantitative PCR reaction mixtures and thermal profiles for the selected genes have previously been described in detail: bacterial 16S rRNA*, nirK*, and *nirS* genes (Schaedler et al. 2018), archaeal 16S rRNA gene (Glodowska et al. 2021), and archaeal *amoA* (Harter et al. 2014) (Table S3).

The primer sets used in this study were adopted from protocols that had been published previously. However, no independent validation of primer coverage, amplification efficiency or taxon-specific performance was conducted for the groundwater microbial communities investigated here. Consequently, qPCR-derived gene copy numbers should primarily be interpreted as relative indicators of gene abundance among samples, rather than as absolute estimates of microbial population size. Additionally, differences in primer coverage and amplification efficiency may impact comparisons between bacterial and archaeal gene abundances.

### Statistical analysis

Data processing and visualization were performed in RStudio Version 2024.12.1+563 using R (version 4.4.2) (R Core Team 2018, Liu et al. 2021). Microbial community analyses were conducted using the microeco package (version 1.15.0) in combination with functions from the vegan package (version 2.7–1).

Prior to multivariate analyses, the ASV table was rarefied to an even sequencing depth (10 185 reads per sample) to account for differences in sequencing effort. The rarefied ASV table was subsequently Hellinger-transformed using the decostand() function (method = “hellinger”) implemented in the vegan package to reduce the influence of highly abundant taxa and account for the compositional nature of amplicon sequencing data.

Collinearity among environmental variables was assessed using variance inflation factors (VIF) calculated from the ordination model. Variables exhibiting high collinearity (VIF > 10) were excluded from further analysis to reduce redundancy and prevent overfitting of the model. The final set of environmental variables included pH, DO, NO_3_⁻, PO_4_^3−^, SO_4_^2−^,and DOC, all of which had acceptable VIF values.

Bray–Curtis distance-based redundancy analysis (db-RDA) was performed to assess the relationship between microbial community composition and environmental variables using the capscale() function implemented via the microeco package. The significance of the overall ordination model and individual environmental variables was evaluated using permutation-based analysis of variance (ANOVA) with 999 permutations via the anova.cca() function. Environmental vectors were fitted to the ordination using envfit() function and visualized in the db-RDA plot.

To visualize overall patterns in microbial community composition independent of environmental constraints, unconstrained ordination analysis was performed using non-metric multidimensional scaling (NMDS) based on Bray–Curtis dissimilarities with the metaMDS() function from the vegan package.

Differences in microbial community composition between sampling sites and filter pore-size fractions were statistically assessed using permutational multivariate analysis of variance (PERMANOVA) implemented with the adonis2() function based on Bray–Curtis dissimilarities and using 999 permutations. Homogeneity of multivariate dispersion among groups was evaluated using the betadisper() function, followed by analysis of variance to determine whether PERMANOVA results were influenced by differences in within-group dispersion.

Principal component analysis (PCA) was performed on standardized geochemical variables (pH, DO, NO_3_⁻, PO_4_^3−^, SO_4_^2−^, and DOC) to explore overall patterns in groundwater hydrochemistry independent of microbial community composition. Prior to the analysis, variables with zero variance (e.g. NH_4_^+^ after data filtering) were excluded. All variables were centered and scaled to unit variance to account for differences in measurement units. PCA was conducted using the prcomp() function and PCA scores were used to visualize hydrochemical gradients and clustering patterns among groundwater samples. In addition, differences in environmental variables between sample groups were assessed using non-parametric Kruskal–Wallis tests or ANOVA, depending on data distribution.

Shannon diversity indices were calculated from the rarefied ASV table to quantify alpha diversity across samples. Differences in diversity among sampling sites were first assessed using the Kruskal–Wallis rank-sum test, followed by pairwise post-hoc comparisons. To evaluate differences between hydrogeological zones (recharge vs anoxic), a Wilcoxon rank-sum test was applied due to the non-normal distribution of Shannon diversity values, as confirmed by Shapiro–Wilk tests. Effect sizes for pairwise comparisons were calculated using the rank-biserial correlation to quantify the magnitude of differences between groups. All statistical analyses were performed in R using the rstatix package (version 0.7.2).

Functional profiles were inferred from 16S rRNA gene data using the Functional Annotation of Prokaryotic Taxa (FAPROTAX v1.2.10), which maps prokaryotic taxa to metabolic functions based on curated literature describing cultured representatives (Louca et al. 2016, Sansupa et al. 2021). Functional assignment coverage was quantified as the proportion of ASVs and sequencing reads assigned to at least one FAPROTAX function. Thus, functional predictions represent potential metabolic capabilities inferred from taxonomy and do not directly reflect gene abundance or metabolic activity.

Predicted functional profiles were correlated with measured geochemical parameters using Spearman’s rank correlation coefficient, implemented in the microeco package. *P* values were adjusted for multiple testing using the Benjamini–Hochberg correction. The correlation patterns were visualized as heatmaps, where only statistically significant correlations were highlighted (*0.01 < *P* ≤ 0.05, **0.001 < *P* ≤ 0.01, ***P ≤ 0.001).

Differences in 16S rRNA gene abundances, predicted functional profiles, and gene copy numbers between sample groups were assessed using Welch’s t-test or the Wilcoxon rank-sum test (wilcox.test() function), depending on data distribution.

Additionally, Random Forest (rf) analysis was performed to identify the microbial genera that best discriminated between predefined sample groups based on their community compositions. Samples were grouped according to sampling sites (wells and karstic spring), which represent distinct environmental conditions within the aquifer system. The microeco package was used to implement rf analysis, with genus-level relative abundance data serving as the predictor variables and sample group identity as the response variable. The importance of each taxon was evaluated using MeanDecreaseGini scores, which estimate its contribution to classification performance based on the extent to which it decreases node impurity across the decision trees. Therefore, higher MeanDecreaseGini scores indicate taxa with greater discriminatory power among sample groups. Sampling replicates were retained as replicate observations within each group and were not treated as independent site categories. Consequently, taxa identified by the rf model are interpreted as features that discriminate between specific hydrogeochemical conditions (wells and karstic spring) rather than as biomarkers that are unique to individual wells (Yatsunenko et al. 2012, Beck and Foster 2014, Martinez-Taboada and Redondo 2020).

## Results

### Hydrogeochemical characteristics of the groundwater at the catchment of the Ammer River (SW Germany)

Pumping rates (Q) in the monitoring wells located in the recharge zone of the catchment were, on average, lower than in the wells located in the anoxic zone and varied from 0.01 to 0.16 l/s. In the anoxic zone, the pumping rate varied from 26 to 60 l/s. Concentrations of O_2_, NO_3_⁻, and DOC varied from 2.0–9.4 mg/l, 5.4–45.4 mg/l and 1.2–11.5 mg/l in the recharge zone to < 0.1–2.0 mg/l, 1–14 mg/l and 0.8–1.0 mg/l in the anoxic zone, respectively. Concentrations of SO_4_^2−^ varied from 10 to 198 mg/l in the recharge zone and from 105 to 186 mg/l in the anoxic zone of the aquifer. Dissolved Fe was detected only in the groundwater sampled from site Has5 (39.9 µg/l) and site TBBrtz (135 µg/l). The concentration of bicarbonate anion was high in all groundwater samples and varied from 382 to 496 mg/l. The pH (from 6.7 to 7.9) of the groundwater showed no clear trends (Table 1).

**Table 1:** Hydrogeochemical characteristics of the groundwater sampled from monitoring wells, a karstic spring and production wells located in the unconfined (recharge zone) and confined (anoxic zone) parts of the carbonate rock aquifer of the Ammer River catchment (SW Germany). Sampling dates are given in Table S1. NA—analyte was not quantified.

Field site	Altitude (m asl)	Pump. rate (Q; l/s)	EC (µS/cm)	pH	DO (mg/l)	NO_3_⁻ (mg/l)	PO_4_^3^⁻ (mg/l)	Cl⁻ (mg/l)	SO_4_^2^⁻ (mg/l)	HCO_3_⁻ (mg/l)	DOC (mg/l)	Fe (µg/l)	H_2_ (ppm)	N_2_O (ppm)
**Sul4**	547.1	0.1	616	7.9	9.4	25.8	0.07	4.8	10.4	382	2.2	NA	2.2	1.8
**Sul3**	510.9	0.01	945	7.1	7.4	9.5	0.24	38.4	198.1	450.9	11.5	<10	NA	NA
**Sul1**	576.0	0.06	518	7.3	8.5	15.4	0.007	5.8	23	376	1.5	<10	1.6	2.2
**Moz4**	545.7	0.10	912	7.6	4.5	24.5	0.030	50.1	138	394	1.0	<10	1.5	9.6
**Moz3**	553.5	0.16	783	7.4	4.3	15.0	0.084	13.1	174	318	3.9	<10	1.2	18.4
**Moz1**	519.2	0.1	641	7.4	7	5.4	0.02	49.2	141.8	225.2	4.1	0	NA	NA
**Has5**	490.7	0.04	761	7.6	9.4	45.4	0.06	20.3	27.1	408	10.3	39.9	13.5	104.6
**Has4**	469.3	0.07	803	6.7	2	23.7	0.02	22.1	143	422.2	1.5	<10	NA	NA
**Has3a**	447.4	0.10	792	7.3	2.5	22.3	0.047	19.3	98	422	2.6	<10	2.1	212.0
**AMQ**	403	50	795	7.4	8.3	42.1	<LOD	29.6	52.6	496	1.2	<10	NA	NA
**TBAlt3**	377	60	828	7.4	3.1	11.7	<0.005	45.6	105	473.5	0.9	<10	NA	NA
**TBBrtz**	395.7	26	711	7.3	0.4	1.1	<0.005	18.5	110	440.5	1	135	<0.5	<0.5
**TBEnt2**	365	35	806	7	0.6	3	<0.005	33.7	124.6	450.9	0.8	<10	<0.5	0.6
**TBPol2**	350	50	934	7.3	1.6	14.3	<0.005	35.4	186.6	475	1	<10	<0.5	45.9

Dissolved gas concentrations differed markedly between sites in the recharge and anoxic zones. In the recharge zone (Sul4, Sul1, Moz4, Moz3, Has5, and Has3a), H_2_ concentrations ranged from 1.2 to 13.5 ppm. N_2_O concentrations varied widely, from 1.8 ppm at Sul4 to over 200 ppm at Has3a, which indicates denitrification activity. In contrast, H_2_ was not determined in the anoxic zone (<0.5 ppm at all measured sites), but there was a large variation in N_2_O. TBEnt2 contained only trace amounts of N_2_O (0.6 ppm). However, TBPol2 showed a pronounced N_2_O accumulation (45.9 ppm) (Table 1). Methane was not detected at all sites (LOQ = 0.5 ppm).

**Figure 2:**
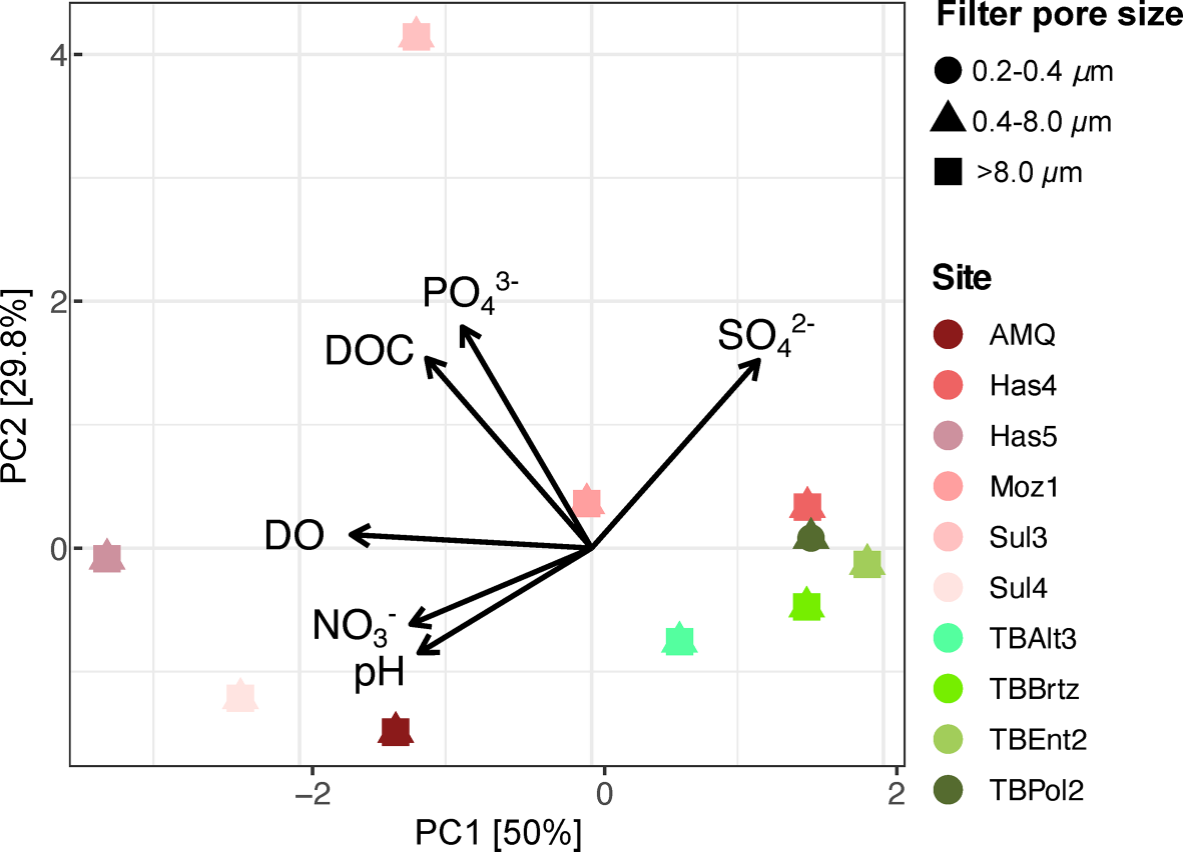
Principal component analysis (PCA) of standardized groundwater geochemical variables (pH, DO, NO_3_^−^, PO_4_^3−^, SO_4_^2−^, and DOC) showing hydrochemical gradients and clustering patterns among groundwater samples. Variables with zero variance were excluded prior to analysis, and all remaining variables were centered and scaled to unit variance to account for differences in measurement units. Generated in RStudio Version 2024.12.1+563 (R version 4.4.2) with the stats package applied.

PCA of the geochemical data revealed clear gradients in groundwater chemistry along the aquifer transect (Fig. 2). The first two principal components explained a substantial proportion of the total variance (PC1: 49.97%, PC2: 29.82%), together accounting for 79.79% of the variability in the dataset. Samples from the recharge and anoxic zones tended to separate along the principal components, reflecting the differences in their hydrochemical conditions. PC1 was primarily associated with DO, NO_3_⁻, pH, and DOC (all negatively correlated), and SO_4_^2−^ (positively correlated), representing a gradient from oxic, NO_3_⁻-rich conditions toward more reduced, SO_4_^2−^-influenced groundwater. In contrast, PC2 was mainly driven by PO_4_^3−^, DOC, and SO_4_^2−^ (all positively correlated), indicating additional variation related to nutrient availability and geochemical heterogeneity within the aquifer.

### Taxonomic composition of groundwater microbial communities

**Figure 3:**
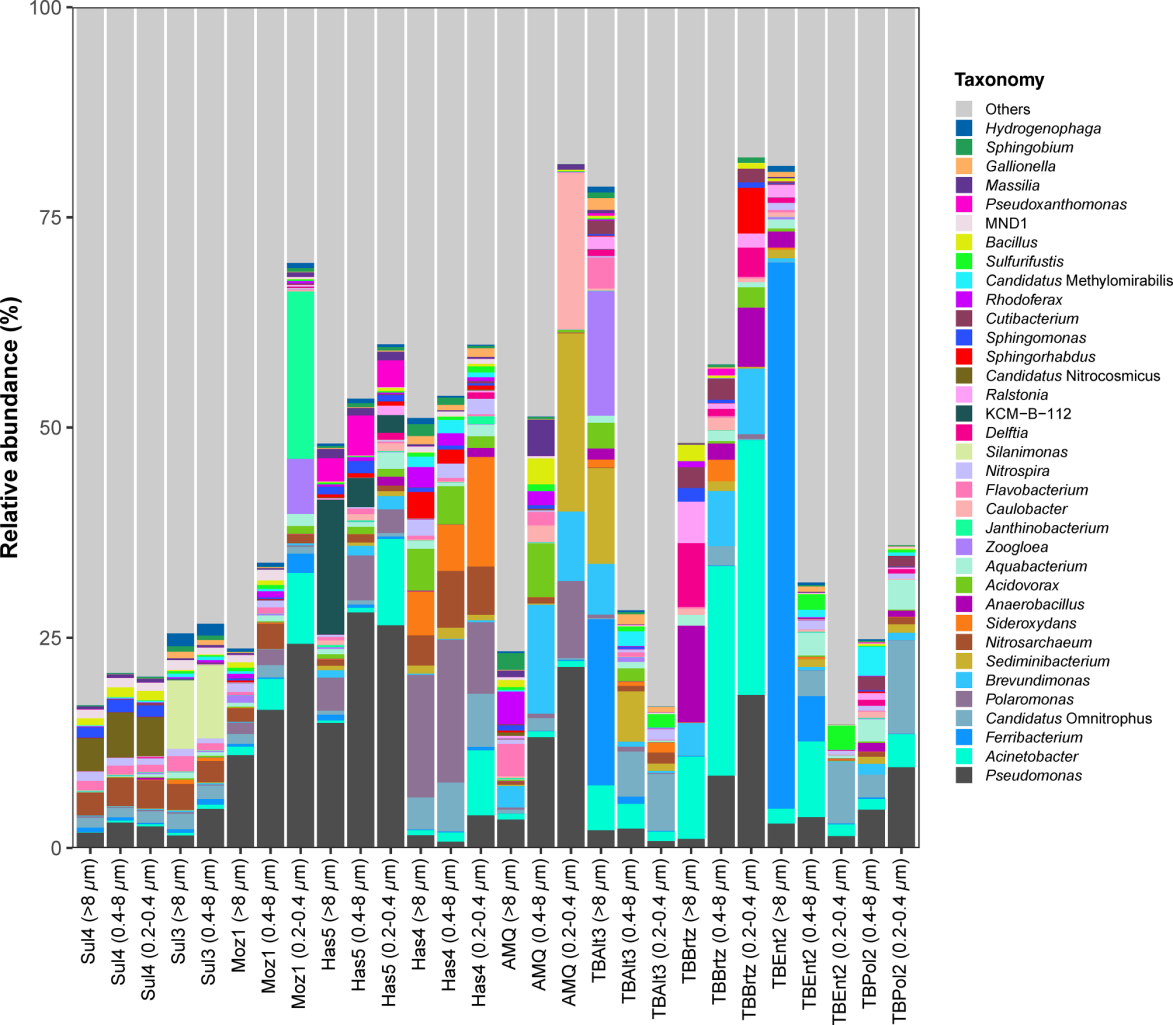
Relative abundance of microbial genera identified in particle-associated (>8 µm) and planktonic (0.4–8 µm and 0.2–0.4 µm) fractions of groundwater microbial communities sampled from field sites located along the Ammer River catchment (SW Germany). The figure shows 35 genera with >1% of relative abundance in at least one sample. Generated in RStudio Version 2024.12.1+563 (R version 4.4.2) with the microeco package applied.

We identified 16S rRNA gene-based relative abundances of microbial taxa in the particle-associated (>8 µm size) and planktonic (0.2–0.4 µm and 0.4–8.0 µm size) fractions of groundwater microbial communities at 10 sampling sites (Fig. 3, Fig S1). Shannon diversity exhibited significant variation across the sampling sites (Kruskal–Wallis test: H = 69.81, *P* = 1.19 × 10^−5^), indicating substantial heterogeneity in microbial diversity within the groundwater system and confirming site identity as an important structuring factor (Fig. S2).

However, pairwise post-hoc comparisons revealed that most site and filter combinations were not statistically significant after false-discovery rate correction (Table S4). Only a limited number of comparisons, primarily those involving the TBBrt, TBAlt3, and TBEnt2 sites, showed marginal differences before adjustment (raw *P* = 0.0495, adjusted *P* = 0.113). These results suggest that the observed variation at the site level is driven by a small subset of contrasts rather than consistent separation across all sites.

Within-site differences related to filter pore size were generally weak and not statistically significant after correction for multiple testing. Only a few comparisons, such as TBEnt2 (0.2–0.4 µm vs >8 µm) and Moz1 (0.2–0.4 µm vs larger fractions), showed marginal effects (raw *P* = 0.0495, adjusted *P* = 0.113). These results indicate that particle size fractionation alone does not strongly influence Shannon diversity across the system.

Relative abundances of specific taxa represented averages of triplicate biological samples of planktonic and particle-associated fractions, except for planktonic samples at sites Sul4 (0.2–0.4 µm), Has5 (0.2–0.4 µm), AMQ (0.2–0.4 µm), AMQ (0.4–8.0 µm size), and particle-associated samples at sites Has5 (>8 µm) and AMQ (>8 µm), for which only two successful extractions were possible (Fig. 3). The DNA extracted from the 0.2–0.4 µm, 0.4–8.0 µm, and >8 µm size fractions was sequenced and analyzed separately without being pooled before analysis. It is important to note that relative abundances were not normalized for 16S rRNA gene copy number variation or primer bias. These factors have the potential to influence the apparent abundance of some taxa, such as *Pseudomonas*. Therefore, relative abundances should be interpreted cautiously as they may not necessarily reflect absolute cell numbers.

NMDS ordination based on Bray–Curtis dissimilarities revealed distinct clustering patterns according to both sampling site and filter size fraction (Fig. S3). The production well samples (TBPol2, TBEnt2, TBBrtz, and TBAlt3) displayed comparatively larger distances among samples, indicating higher variability in microbial community composition across these sites. In contrast, samples from monitoring wells (Sul4, Sul3, Moz1, Has5, and Has4) formed more compact clusters with lower inter-sample distances, suggesting more homogeneous microbial communities within this group.

Samples from the karst spring AMQ showed a pronounced separation of the 0.2 µm size fraction relative to the larger pore-size fractions, a pattern that was less evident in monitoring wells. The microbial community detected in the Ammer spring can be distinct from those observed in individual groundwater wells. The spring integrates groundwater from multiple upstream sources and depths, and therefore likely represents a combined microbial assemblage of the aquifer. Within production wells, the 0.2 µm and 0.4 µm fractions generally clustered separately from the 8 µm fraction, indicating differences between smaller free-living microbial communities and larger particle-associated assemblages. An exception was observed for the 0.2 µm fraction of TBBrtz, which clustered more closely with the larger size fractions.

PERMANOVA analysis confirmed that microbial community composition differed significantly among sampling sites (R^2^ = 0.45, *P* ≤ 0.001) and among filter size fractions (R^2^ = 0.08, *P* ≤ 0.001), with the overall model explaining 53% of the observed variation in community structure. However, analysis of multivariate dispersion revealed significant heterogeneity among sites (PERMDISP, *P* ≤ 0.001), indicating that differences in community composition were influenced not only by centroid separation but also by differences in within-group variability (Fig. S4).

The primary discriminatory genera among groundwater microbial communities were identified using rf analysis based on MeanDecreaseGini importance scores (Fig. S5). Samples were grouped according to field site and associated hydrogeological conditions. Microbial communities from Sul4 were primarily characterized by the MND1 group, *Sphingomonas, Bacillus*, and *Candidatus* Nitrocosmicus, whereas Sul3 communities were associated with *Aquicella, Polycyclovorans*, and *Hydrogenophaga*. Moz1 communities were characterized by the pLW-20 group and *Methyloglobulus*. In Has5, *Pseudoxanthomonas, Pseudomonas*, and *Thiobacillus* showed high discriminatory importance, while Has4 communities were associated with *Nitrospira, Nitrosarchaeum, Sideroxydans, Polaromonas, Bdellovibrio, Candidatus* Methanoperedens, and *Candidatus* Omnitrophus. AMQ communities were characterized by *Brevundimonas* and *Caulobacter*, whereas TBAlt3 was associated with *Gallionella* and *Sediminibacterium*. TBBrtz communities were primarily characterized by *Anaerobacillus, Ochrobactrum*, and Acinetobacter, while TBEnt2 communities were associated with *Sulfurifustis, Ferribacterium*, and *Desulfosporosinus*. Finally, *Candidatus* Methylomirabilis and *Candidatus* Tenderia showed high discriminatory importance in TBPol2 communities.

16S rRNA gene sequencing results showed that *Pseudomonas* was one of the most abundant taxa at the genus level identified along the aquifer transect. The relative abundance of *Pseudomonas* was slightly higher in the planktonic fractions of the groundwater microbial communities. In groundwater samples collected from Moz1, Has5, AMQ, TBBrtz, and TBPol2, the relative abundance of *Pseudomonas* was typically above 10%. In addition, other genera were also found to be abundant (>2%) in the majority of size fractions at sampling sites located in the recharge zone and anoxic zone of the aquifer. These genera included *Brevundimonas* (sites: AMQ, TBAlt3, TBBrtz), Acinetobacter (sites: Moz1, Has5, Has4, TBalt3, TBBrtz, TBEnt2, TBPol2), *Sediminibacterium* (sites: AMQ, TBAlt3), *Sideroxydans* (sites: Has4 and TBBrtz), *Aquabacterium* (Sites: Has5, TBEnt2, TBPol2), *Sphingorhabdus* (sites: Has4 and TBBrtz), *Acidovorax* (Sites: AMQ, Has4, TBAlt3), *Candidatus* Omnitrophus (sites: Has4, TBAlt3, TBBrtz, TBEnt2, TBPol2), and *Flavobacterium* (sites: AMQ and TBAlt3) (Fig. 3, Fig. S1, Fig. S5).

Other genera typically showed a tendency to dominate either in the recharge zone or the anoxic zone of the aquifer. For example, we observed higher relative abundances of the following genera in the recharge zone: *Candidatus* Nitrocosmicus (site: Sul4), *Nitrosarchaeum* (sites: Sul4, Sul3, Moz, Has4), *Polaromonas* (sites: Has4, Has5, AMQ), *Rhodoferax* (sites: AMQ and Has4), *Massilia* (site: AMQ), etc. In contrast, the following genera were more abundant in the anoxic zone: *Anaerobacillus* (sites: TBBrtz and TBEnt2), *Cutibacterium* (sites: TBBrtz and TBPol2), *Sulfurifustis* (site: TBEnt2), *Methylobacterium* (previously *Methylorubrum*, family Methylobacteriaceae) (site: TBPol2), *Allorhizobium***-***Neorhizobium***-***Pararhizobium***-***Rhizobium* (family *Rhizobiaceae*; site: TBBrtz), *Ferribacterium* (mainly at sites TBAlt3 and TBEnt2, but also 2.72% in the 0.2–0.4 µm size fraction of Moz1), *Candidatus* Brocadia (site: TBEnt2), *Ralstonia, Ochrobactrum*, and *Lysinibacillus* (site: TBBrtz), among others (Fig. 3, Fig. S1, Fig. S5).

### Abundance of bacterial, archaeal, and N cycle-specific genes in the groundwater microbial community

The qPCR analysis was used to quantify absolute gene copy numbers of bacterial and archaeal 16S rRNA genes, thereby complementing the 16S rRNA gene amplicon sequencing approach, which provides relative taxonomic abundances based on sequence proportions and comparison to reference databases. Although both approaches provided different types of quantitative information, both are subject to methodological biases.

**Figure 4:**
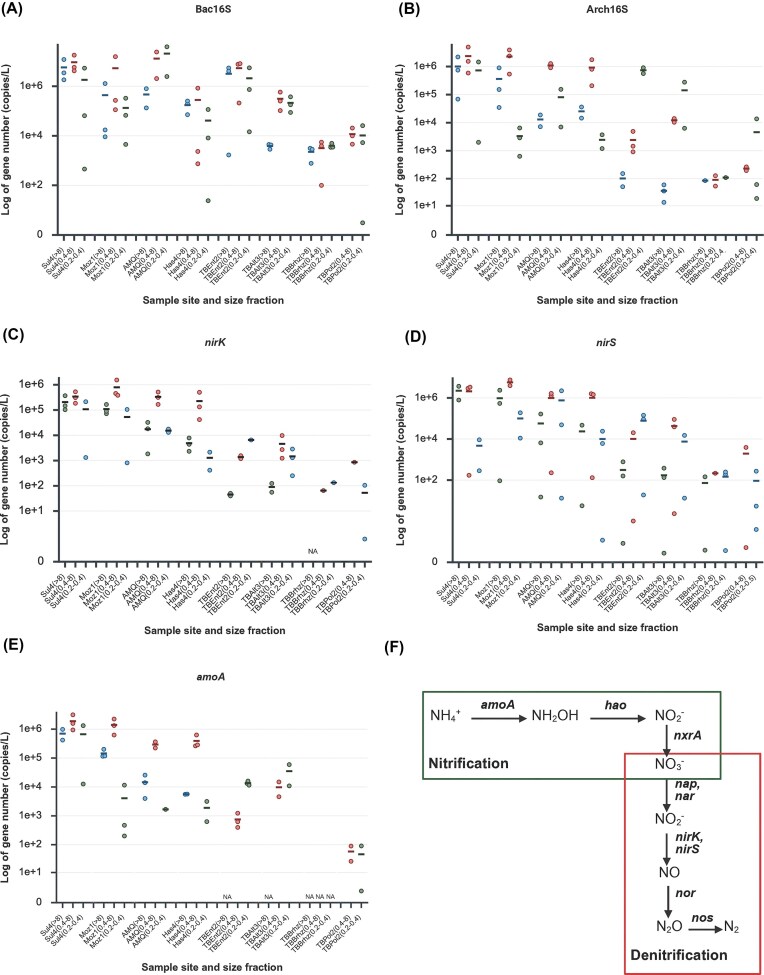
Abundance of(A) bacterial 16S rRNA genes, (B) archaeal 16S rRNA genes, (C) *nirK*, (D) *nirS*, and (E) archaeal *amoA* involved in (F) nitrification and denitrification in particle-associated (>8 µm) and planktonic (0.4–8.0 µm and 0.2–0.4 µm) fractions of groundwater microbial communities sampled from field sites along the Ammer River catchment (SW Germany). The figure shows the log of gene copies (mean values) quantified in 1 L of groundwater. Created in BioRender. Abramov, S. (2026) https://BioRender.com/6jvzr5t.

Results from qPCR analysis showed that measured bacterial 16S rRNA gene copy numbers generally exceeded measured archaeal 16S rRNA gene copy numbers in groundwater from the recharge area of the aquifer (unconfined sites: Sul4, Moz1, and AMQ) (Fig. 4). However, there were exceptions. In the well at site Moz1, archaeal 16S rRNA gene copy numbers were supposedly equal to bacterial 16S rRNA gene copy numbers in the particle-associated (>8 µm size; 3.61 × 10^5^ copies/l vs 4.37 × 10^5^ copies/l, respectively) and planktonic (0.4–8.0 µm size; 2.27 × 10^6^ copies/l vs 5.34 × 10^6^ copies/l, respectively) fractions. Because bacterial and archaeal 16S rRNA genes were quantified using different primer sets and qPCR assays, these values should be interpreted as approximate indicators of relative gene abundance rather than direct comparisons of microbial population size.

The presence of genes associated with nitrite reductases (*nirK, nirS*) and ammonia monooxygenase (*amoA*) was identified in all sampled locations within the recharge zone of the aquifer, exhibiting a range from ∼10^4^ to ∼10^6^ copies/l. However, in the planktonic 0.2–0.4 µm size fraction at the Moz1 and AMQ sites, the number of *amoA* gene copies was ∼10^3^ copies/l of groundwater. Similarly, in the planktonic 0.2–0.4 µm size fraction at site Sul4, the number of *nirS* gene copies was also ∼10^3^ copies/l.

In the anoxic zone of the aquifer (sites: Has4, TBAlt3, TBBrtz, TBEnt2, TBPol2), measured copy numbers of bacterial and archaeal 16S rRNA genes were significantly lower (Wilcoxon rank-sum test: W = 103, 0.01 < *P* ≤ 0.05 and W = 118, *P* ≤ 0.001, respectively) than in the recharge zone and generally did not exceed 10^6^ copies/l of groundwater, except for bacterial planktonic fractions (0.2–0.4 and 0.4–8.0 µm; size 2.1 × 10^6^ ± 3.0 × 10^6^ and 5.37 × 10^6^ ± 4.5 × 10^6^ copies/l, respectively) and particle-associated fractions (>8 µm size, 3.2 × 10^6^ ± 2.8 × 10^6^ copies/l) at site TBEnt2 (Fig. 4, Fig. S6A-B).

Within the anoxic zone, measured bacterial 16S rRNA gene copy numbers were supposedly higher than measured archaeal 16S rRNA gene copy numbers (Wilcoxon rank-sum test: W = 123.5, 0.01 < *P* ≤ 0.05) (Fig. 4, Fig. S6C). However, in the planktonic 0.45–8.0 µm size fraction at the Has4 site, archaeal 16S rRNA gene copy numbers were 9.2 × 10^5^ ± 7.8 × 10^5^ copies/l, while bacterial 16S rRNA gene copy numbers were 2.8 × 10^5^ ± 4.8 × 10^5^ copies/l. Because bacterial and archaeal qPCR assays rely on different primer sets and amplification efficiencies, these values should be interpreted as approximate, assay-dependent indicators rather than direct quantitative comparisons between domains.

Copy numbers of *nirK, nirS*, and *amoA* genes were significantly lower in the anoxic zone of the aquifer (Wilcoxon rank-sum test: W = 115, *P* ≤ 0.001; W = 122, *P* ≤ 0.001; and W = 95, *P* ≤ 0.01, respectively; Fig. 4, Fig. S6D–F). We did not detect *amoA* genes in the groundwater sampled from site TBBrtz, nor in the particle-associated (>8 μm size) fractions of the groundwater sampled from sites TBAlt3 and TBEnt2. Furthermore, *nirK* was not detected in the particle-associated fraction (>8 µm size) at site TBBrtz.

It should be noted that 16S rRNA gene copy numbers do not directly correspond to microbial cell counts because different taxa may contain different numbers of rRNA operons. Consequently, the reported values represent gene copy numbers per groundwater volume rather than direct estimates of microbial population size. In addition, primer mismatches and differences in amplification efficiency may influence the quantification of specific taxonomic groups, including archaeal lineages such as *Nitrososphaeria*.

### Comparative analysis of particle-associated and planktonic microbial communities

**Figure 5:**
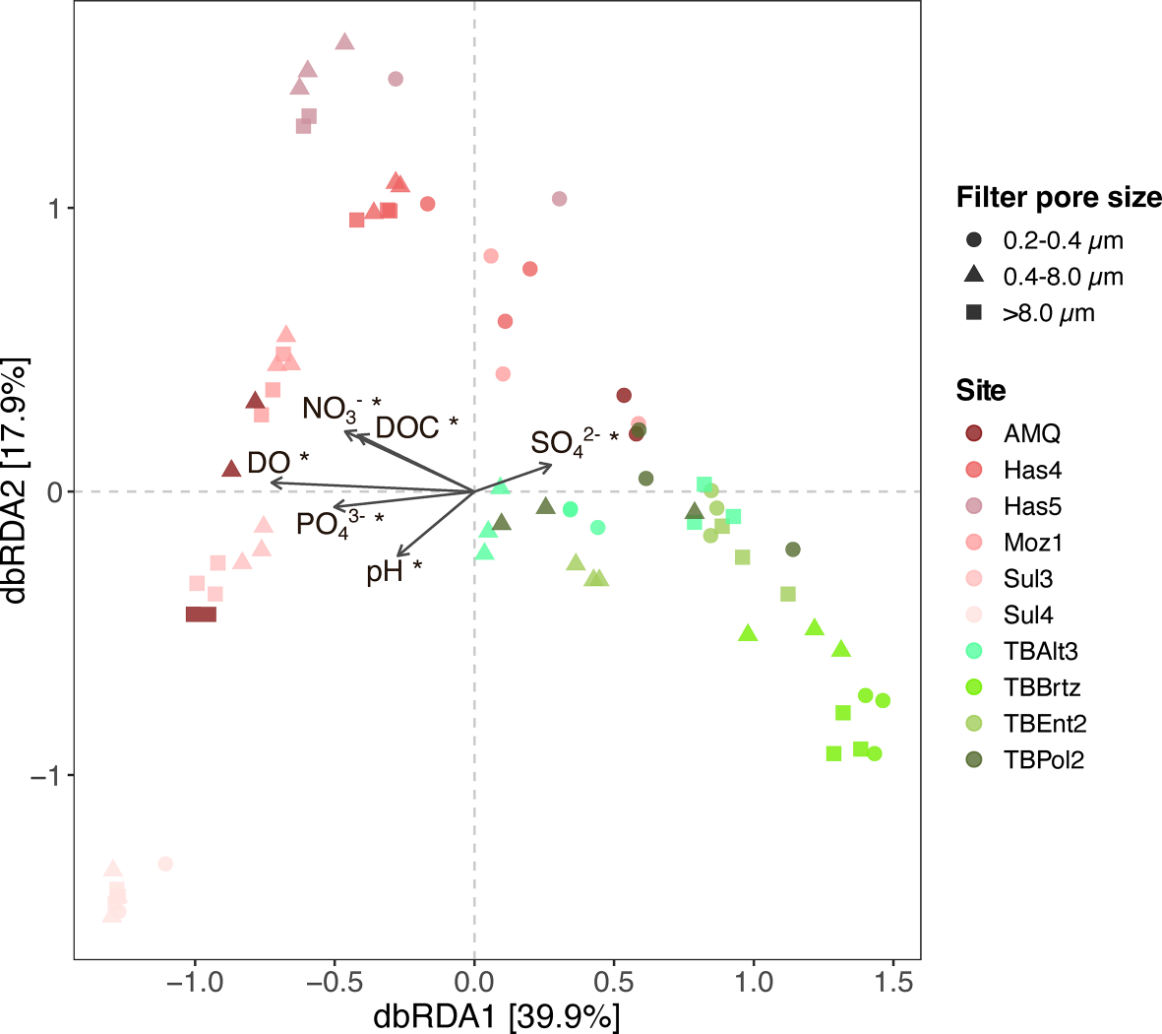
Bray–Curtis distance-based redundancy analysis (db-RDA) biplot illustrating the relationship between microbial community composition (at the Genus level) and key physicochemical parameters across different size fractions of groundwater microbial communities from 10 field sites at the Ammer River catchment (SW Germany). Environmental vectors represent the influence of pH, dissolved oxygen (DO), NO_3_⁻, PO_4_^3^⁻, SO_4_^2^⁻, and DOC on microbial distribution. Asterisks (*) indicate environmental variables with a statistically significant effect on microbial composition. Statistical significance of environmental variables was assessed using a permutation-based ANOVA. Arrow direction indicates the gradient of increasing variable values, while arrow length represents the strength of the variable’s effect. The angle between arrows reflects relationships between variables, with acute angles suggesting positive correlations and opposing arrows indicating negative correlations. Samples positioned along an arrow are strongly influenced by the corresponding environmental variable. Microbial communities were categorized into particle-associated (>8 µm) and planktonic fractions (0.4–8 µm and 0.2–0.4 µm), showing distinct clustering patterns driven by hydrochemical conditions. Generated in RStudio Version 2024.12.1+563 (R version 4.4.2) with the microeco package applied.

Comparative analysis of the microbial communities using distance-based redundancy analysis (db-RDA; Fig. 5) revealed clear and distinct patterns. Specifically, the microbial communities in the recharge area (represented by warm-coloured markers in Fig. 5) differed markedly from those in the anoxic zone (represented by green-coloured markers in Fig. 5). The observed clustering patterns in the db-RDA ordination were supported by PERMANOVA analysis, confirming that microbial community composition differed significantly among sampling groups (*P* ≤ 0.001).

In the recharge area, we observed that the particle-associated and planktonic size fractions of the groundwater microbial communities were generally similar. However, the planktonic 0.2–0.4 µm size fraction of the Ammer spring water differed from the 0.4–8.0 µm and >8 µm size fractions. A similar pattern was observed for the Moz1 groundwater size fractions.

In the anoxic zone, the >8 µm particle-associated size fraction of the groundwater microbial community from TBAlt3 was distinct from both planktonic fractions. In addition, the 0.2–0.4 µm planktonic size fraction of the groundwater microbial community from TBBrtz differed from the 0.4–8.0 µm size fraction.

**Figure 6:**
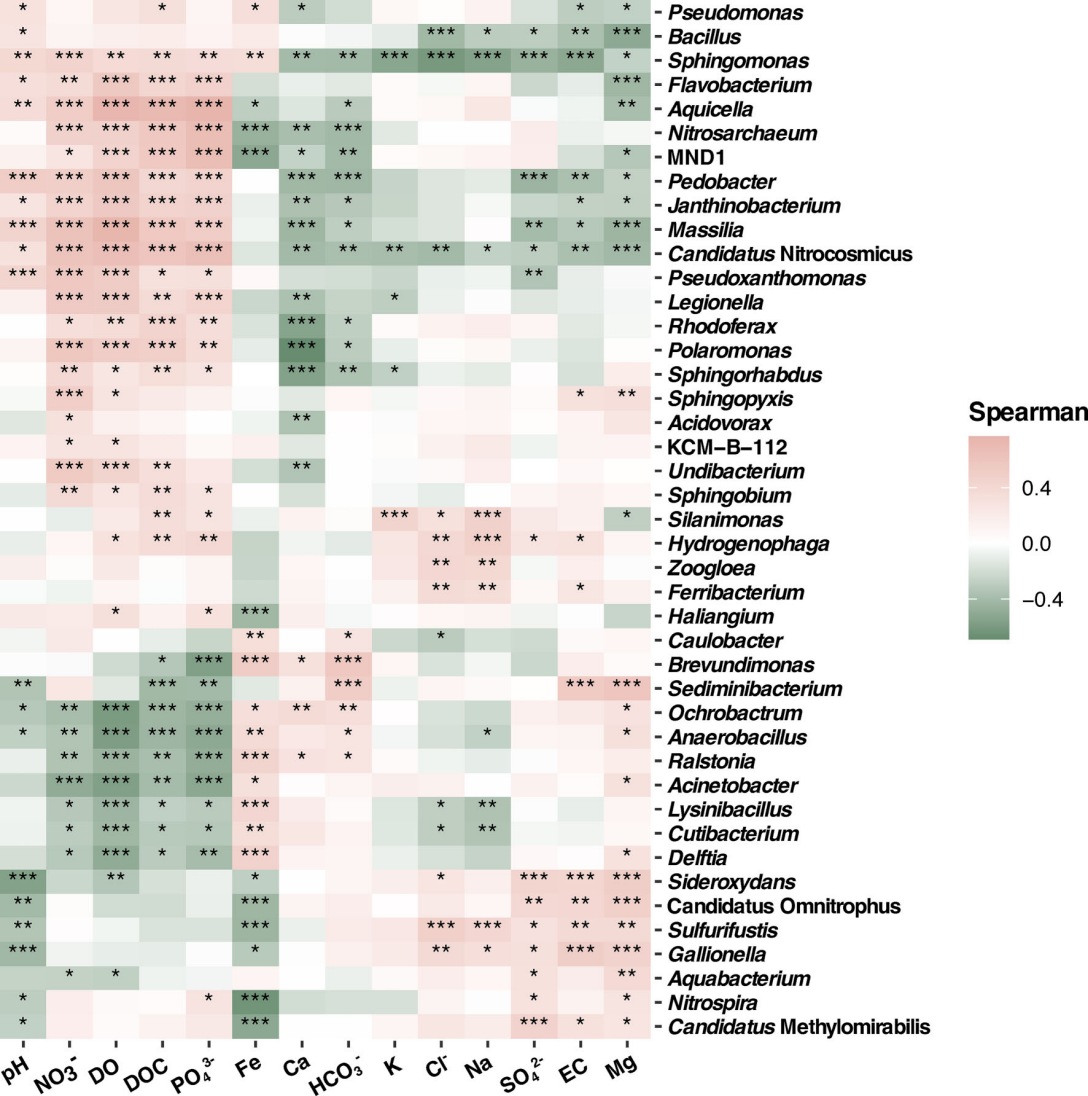
Correlation heatmap showing relationships between the top 43 most abundant groundwater microbial taxa and key biogeochemical parameters. Microbial communities were analyzed in particle-associated (>8 µm) and planktonic (0.4–8 µm and 0.2–0.4 µm) fractions, sampled from wells and springs along the Ammer River catchment transect. Correlations were assessed using Spearman’s rank correlation coefficient, with significance adjusted using the Benjamini–Hochberg correction. Statistical significance is denoted as follows: *0.01 < *P* ≤ 0.05, **0.001 < *P* ≤ 0.01, ***P ≤ 0.001. Generated in RStudio Version 2024.12.1+563 (R version 4.4.2) with the microeco package applied.

Correlation analysis (Spearman’s rank correlation coefficient and Benjamini–Hochberg correction) revealed significant positive correlation between concentration of NO_3_⁻ and KCM-B-112 (0.01 < *P* ≤ 0.05), *Pseudoxanthomonas* (*P* ≤ 0.001), *Acidovorax* (0.01 < *P* ≤ 0.05), *Hydrogenophaga* (0.01 < *P* ≤ 0.05), *Legionella* (*P* ≤ 0.001), *Massilia* (*P* ≤ 0.001), *Undibacterium* (*P* ≤ 0.001), *Rhodoferax* (0.01 < *P* ≤ 0.05), *Polaromonas* (*P* ≤ 0.001), and *Aquicella* (*P* ≤ 0.001). In addition, *Pseudoxanthomonas* (*P* ≤ 0.001 and 0.01 < *P* ≤ 0.05, respectively), *Massilia* (*P* ≤ 0.001 and *P* ≤ 0.001, respectively), and *Aquicella* (*P* ≤ 0.001 and *P* ≤ 0.001, respectively) were positively correlated with DO and DOC concentrations. *Anaerobacillus* (0.001 < *P* ≤ 0.01), *Lysinibacillus* (0.01 < *P* ≤ 0.05), *Ralstonia* (0.001 < *P* ≤ 0.01), *Aquabacterium* (0.01 < *P* ≤ 0.05) and *Acinetobacter* (*P* ≤ 0.001) significantly correlated with a decrease in NO_3_⁻ concentration in the groundwater (Fig. 6).

### Functional prediction of particle-associated and planktonic microbial communities

The functional profiling of the microbial communities was performed using the FAPROTAX database (Louca et al. 2016, Sansupa et al. 2021). This approach was used to infer potential metabolic functions, based on taxonomic composition. The results indicated a high chemoheterotrophic potential (from 17.19% to 60%; Table S5). FAPROTAX assigned at least one putative metabolic function to 38.4% of ASVs, representing 36.5% of total sequencing reads, with sample-level assignment coverage ranging from 15.1% to 71.8%. It should be noted that these predictions represent inferred metabolic potential rather than directly measured activity, as no eco-physiological experiments were conducted. Therefore, all functional interpretations are described here as potential activities. In addition, shotgun metagenomic sequencing would provide a more direct assessment of microbial functional potential. However, the present study was designed as a 16S rRNA gene amplicon survey with a focus on the composition of microbial communities. In several cases, the recovered DNA material was limited and thus was optimized for amplicon sequencing. Consequently, metagenomic sequencing was not within the focus of the present study.

The overall distribution of metabolic functions was relatively uniform along the aquifer transect. For example, there were no statistical differences in chemoheterotrophic potential (Wilcoxon rank-sum test: W = 103, *P* = 0.877; Fig. S7A), aerobic chemoheterotrophic potential (Wilcoxon rank-sum test: W = 109, *P* = 0.674; Fig. S7B), and anaerobic chemoheterotrophic potential (Wilcoxon rank-sum test: W = 58, *P* = 0.068; Fig. S7C) in recharge and anoxic zones. In addition, we observed a comparatively high relative abundance of micro-organisms potentially involved in nitrification (from 0% to 2.78%) in all the wells of the recharge zone and in sites TBAlt3 and TBEnt2 (especially in the 0.2–0.4 µm size fraction in the anoxic zone) (Table S5).

**Figure 7:**
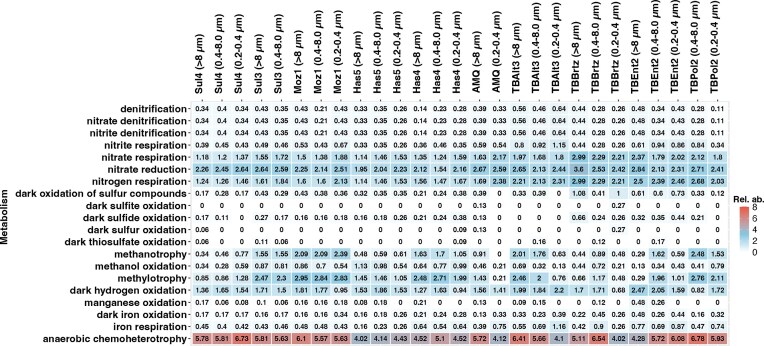
Functional predictions (selected functions) of particle-associated (>8 µm) and planktonic (0.4–8 µm and 0.2–04 µm) fractions of groundwater microbial communities sampled from field sites located along the Ammer River catchment (SW Germany). The complete functional prediction is shown in Table S2. Functional predictions were performed using FAPROTAX v1.2.10 (Louca et al. 2016, Sansupa et al. 2021). Predicted metabolic potential in FAPROTAX represents the summed relative abundance of ASVs assigned to taxa with experimentally verified metabolic functions. Generated in RStudio Version 2024.12.1+563 (R version 4.4.2) with the microeco package applied.

Although the potential for methanogenesis was low between aquifer transects (from 0% to 0.47%) (Fig. S7), the potential for methanotrophy and methylotrophy were inferred along the aquifer (from 0% to 4.17% and 0% to 5.73%, respectively) and were not significantly different (Wilcoxon rank-sum test: W = 98, *P* = 0.982 and W = 121, *P* = 0.334, respectively) between recharge and anoxic zones (Fig. 7, Fig. S7D and E).

The potential for complete denitrification with NO_3_⁻ or NO_2_⁻ as electron acceptors was not found to be abundant (from 0% to 2.35%). However, the first step of denitrification and DNRA (nitrate reduction) potential was abundant in the groundwater samples collected along the aquifer transect, but slightly more pronounced (from 0.66% to 7.06%) than the potential for complete denitrification. The maximum relative abundance was found in the anoxic zone of the aquifer, with a relative abundance of 7.06% in the >8 µm particle-associated size fraction of groundwater from site TBBrtz (Fig. 7).

The relative abundance of microorganisms potentially capable of dark (non-photosynthetic) oxidation of sulfur compounds, the oxidation of reduced sulfur compounds (RSCs) in the absence of light, was significantly higher (Wilcoxon rank-sum test: W = 121, 0.01 < *P* ≤ 0.05) in the anoxic zone of the aquifer and varied overall from 0% to 3.53% along the aquifer (Fig. 7, Fig. S7F). While the relative abundance of dark (non-photosynthetic) sulfide oxidation was not significantly different (Wilcoxon rank-sum test: W = 76.5, *P* = 0.32) along the aquifer transect in the recharge and anoxic zones (overall from 0% to 2.35%) (Fig. 7, Fig. S7G). In addition, the groundwater microbial communities were inferred to have the potential for respiration of sulfur compounds (from 0 to 4.55%), sulfate respiration (from 0 to 3.41%) and thiosulfate respiration (from 0 to 1.39%) (Table S5).

The metabolic potentials for dark (non-photosynthetic) iron(II) oxidation (from 0% to 1.33%) as well as iron(III) respiration (from 0% to 1.91%) were significantly higher (Wilcoxon rank-sum test: W = 26.5, 0.001 < *P* ≤ 0.01 and W = 18, *P* ≤ 0 .001, respectively) in the anoxic zone of the aquifer. The highest relative abundance of dark (non-photosynthetic) iron(II) oxidation was found in the anoxic zone (site: TBBrtz) (Fig. 7, Fig. S7H and I).

Another metabolic process that may be of interest in understanding the functioning of the groundwater microbial community is dark (non-photosynthetic) hydrogen oxidation potential (from 0% to 5.04%), which did not differ significantly between recharge and anoxic zones (Wilcoxon rank-sum test: W = 76, *P* = 0.312) (Fig. S7J). Nevertheless, microorganisms potentially capable of this process were present at all sampling sites (Fig. 7), but the highest relative abundance was found at well TBEnt2 (anoxic zone).

**Figure 8:**
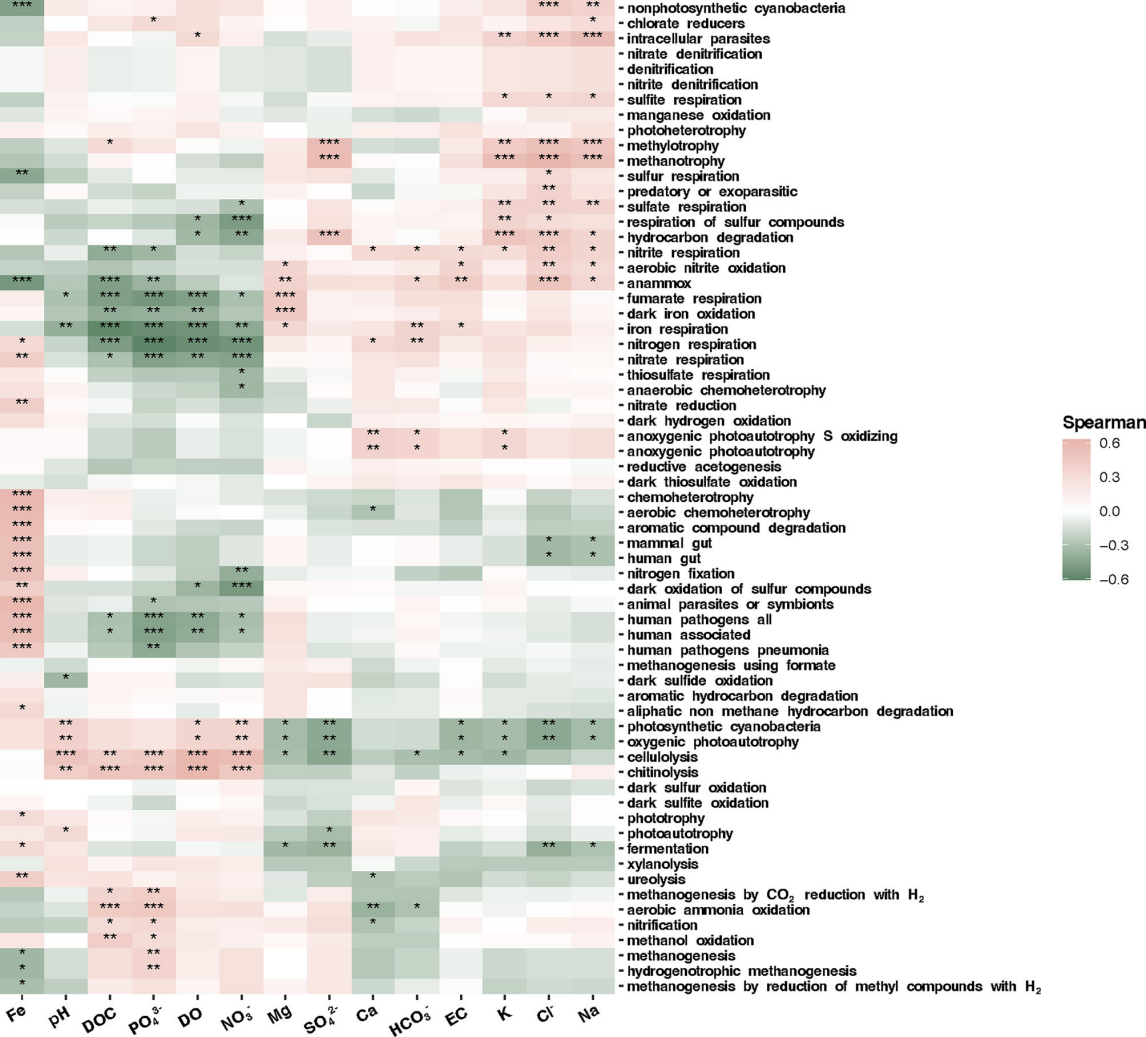
Correlation of predicted metabolic potential and hydrochemical parameters. Particle-associated (>8 µm) and planktonic (0.4–8 µm and 0.2–0.4 µm) fractions of groundwater microbial communities were sampled from wells and springs along the transect of the Ammer River catchment. Correlations were assessed using Spearman’s rank correlation coefficient, with significance adjusted by Benjamini–Hochberg correction. Statistical significance is denoted as follows: *0.01< *P* ≤ 0.05, **0.001< *P* ≤0.01, ***P ≤ 0.001. Functional predictions were performed using FAPROTAX v1.2.10 (Louca et al. 2016). Generated in RStudio Version 2024.12.1+563 (R version 4.4.2) with the microeco package applied.

Correlations between predicted metabolic functions and environmental variables were assessed using Spearman’s rank correlation analysis (Fig. 8). The resulting heatmap revealed significant associations between specific functional potentials and key geochemical parameters, indicating that environmental conditions may influence the distribution of inferred metabolic traits across the aquifer.

We acknowledge the limitations of taxonomy-based functional prediction approaches such as FAPROTAX and note that future studies employing metagenomic sequencing would enable a more direct and comprehensive assessment of the functional potential in the aquifer microbial community.

## Discussion

### Alternative electron donors fuel microbial NO_3_⁻ reduction across the aquifer of the Ammer River

The sampling campaign captured recharge-dominated hydrological conditions typical of late winter to early spring, when elevated groundwater recharge enhances solute transport from surface environments into carbonate aquifers. Such hydrological conditions likely promote the delivery of O_2_, NO_3_⁻, and DOC into the recharge zone and may therefore contribute to the pronounced hydrochemical and microbial gradients observed along the aquifer transect. The observed progressive depletion of O_2_, NO_3_⁻, and DOC together with elevated SO_4_^2^⁻ concentrations suggest a shift from oxidizing to reducing conditions along the flow path.

Despite this overall trend, fluctuations in NO_3_⁻ and DOC concentrations within both zones indicate that recharge-derived inputs remain spatially heterogeneous, likely due to mixing of groundwater with variable hydrochemical signatures (Visser et al. 2021). Due to the low concentration of sedimentary organic carbon (<0.06 mass-%) in the Upper Muschelkalk, the dynamics of the microbial community in the aquifer are likely governed primarily by variations in the external supply of organic electron donors (e.g. DOC) and electron acceptors (e.g. O_2_ and NO_3_⁻), as well as by internal electron donors (e.g. pyrite) (Griebler and Lueders 2009, Akob and Küsel 2011, Yan et al. 2020, Yan et al. 2021).

Nevertheless, the interpretation of groundwater microbial communities should consider potential methodological effects associated with sampling different groundwater outlets, including monitoring wells, production wells, and springs. Pumping-induced mixing and biofilm detachment could influence the relative contribution of planktonic and particle-associated microorganisms, particularly in fractured carbonate aquifers where flow heterogeneity is high. Furthermore, sessile biofilm communities attached to aquifer rock surfaces were not directly sampled and could represent an important reservoir of denitrifying microorganisms.

*Pseudomonas* capable of heterotrophic growth in oxic conditions or NO_3_⁻ reduction in anoxic conditions was consistently detected across multiple sites and size fractions and has been reported previously from groundwater environments. However, because laboratory negative controls were not included in this study, a contribution of methodological bias cannot be fully excluded. Consequently, the abundance of *Pseudomonas* and other individual taxa should be interpreted with caution. At the same time, shallow karst systems are highly vulnerable to surface water intrusion, agricultural runoff and wastewater infiltration (Goldscheider 2005). Therefore, *Pseudomonas* introduced via surface contamination could become part of the groundwater microbial community (Kämpfer et al. 1991, Zhou and Tiedje 1995, Feris et al. 2004, Keller et al. 2024)

*Pseudomonas*, as well as other taxa, could contribute to aerobic chemoheterotrophy. During recharge events, these heterotrophic microorganisms could oxidize DOC from the soil, which enters the aquifer and supports microbial diversity and abundance (Benk et al. 2019). Heterotrophic microorganisms may also contribute to NO_3_⁻ turnover in the anoxic aquifer, where O_2_ is depleted, but DOC or particulate organic carbon (not quantified in this study) remains. Previous studies have shown that a sufficient proportion of organic carbon remains bioavailable in groundwater (Einsiedl et al. 2007, Peter et al. 2012, Shabarova et al. 2014, Shen et al. 2015, Pracht et al. 2018, Wu et al. 2018, Hofmann et al. 2020). The presence of taxa associated with denitrification (e.g. *Pseudomonas, Polaromonas, Ferribacterium*) (Wang et al. 2014, Arat et al. 2015, Tan et al. 2021) or partial denitrification (e.g. *Rhodoferax, Pseudoxanthomonas*) (Chen et al. 2002, Risso et al. 2009, Wang et al. 2014) suggests that the aquifer microbiome retains a substantial potential for denitrification that could be activated following inputs of organic matter. However, a substantial fraction of the groundwater microbiome remained functionally unresolved. Functional predictions based on FAPROTAX should be interpreted cautiously because they infer metabolic potential from taxonomy rather than from direct gene expression or activity. Nevertheless, future metagenomic and metatranscriptomic analyses will help clarify the functional role of uncultured and taxonomically unresolved groundwater microorganisms.

The decline in DOC concentrations toward the anoxic zone is likely to increase the ecological importance of alternative electron donors for NO_3_⁻ reduction. In oligotrophic groundwater conditions, chemolithotrophic denitrification linked to RSCs, Fe(II), H_2_ or potentially CH_4_ could provide an energetically favourable pathway for microbial respiration. The elevated bicarbonate concentrations throughout the aquifer further suggest that autotrophic or mixotrophic carbon fixation could supplement microbial growth in carbon-limited groundwater environments.

The presence of H_2_ in several wells suggests that fermentation or water-rock interactions could provide an additional electron donor source. Low-temperature generation of H_2_ through Fe-bearing mineral alteration has been documented in ultramafic and mafic rocks (Mayhew et al. 2013) and basalts (Huang et al. 2025). The occurrence of *Hydrogenophaga, Ralstonia*, and *Pseudomonas* identified in the recharge zone and anoxic zone, as well as *Ochrobactrum* in the TBBrtz well, could couple H_2_ oxidation with NO_3_⁻ reduction (Tang et al. 2020).

Pyrite-bearing Upper Muschelkalk likely represents one of the most important electron donor reservoirs for autotrophic or mixotrophic NO_3_⁻ reduction in the anoxic aquifer. The elevated SO_4_^2−^ concentrations in the anoxic zone suggest the importance of pyrite-derived RSCs oxidation coupled to NO_3_⁻ reduction. The enrichment of *Sulfurifustis* in the anoxic zone supports the potential importance of RSCs-driven denitrification under low DOC conditions (Kojima et al. 2015).

Fe(II)-mediated denitrification could further contribute to NO_3_⁻ turnover in the aquifer. Pyrite oxidation can release dissolved Fe^2+^, which may subsequently participate in NRFeOx or precipitate as secondary carbonate minerals such as siderite under bicarbonate-rich conditions. Indeed, dissolved Fe was detected only in the groundwater of sites Has5 and TBBrtz. The presence of dissolved Fe in the O_2_-saturated groundwater at site Has5 correlates with a high DOC content and can be explained by the complexation of Fe^2+^ by organic carbon (Suzuki et al. 1992). The occurrence of *Gallionella, Acidovorax*, and *Aquabacterium* suggests that both autotrophic and mixotrophic Fe(II)-oxidizing denitrifiers could be active within the aquifer system (Zhou et al. 2017).

Thus, in the recharge zone, heterotrophic microorganisms are likely to play the major role in NO_3_^⁻^ turnover. In the anoxic zone of the aquifer, DOC is depleted, and microorganisms may use alternative electron donors. In the recharge zone, both particle-associated (>8 µm) and planktonic (0.4–8.0 µm) fractions contributed to high gene abundance, whereas in the anoxic zone, gene abundance often exceeded expectations in larger fractions, such as the particle-associated >8 µm at site TBEnt2. However, gene abundance patterns derived from qPCR should be interpreted as relative indicators of these transitions rather than quantitative measures of process rates or biomass shifts. This suggests that denitrification activity in the anoxic part of the aquifer is likely mainly associated with particle-associated or biofilm-associated microbial communities. Nevertheless, specific hydrogeochemical conditions of the groundwater, such as low O_2_, low DOC and high NO_3_⁻ concentrations, could contribute to the potential coupling of NO_3_⁻ reduction with Fe(II) and RSCs oxidation.

Overall, the NO_3_⁻ turnover in the aquifer may be mainly supported by heterotrophic denitrifiers in recharge zones, while chemolithotrophic pathways could become increasingly important in the anoxic zone under DOC-limited conditions. This transition likely reflects a shift from surface-derived organic carbon as the main electron donor to lithology-derived reduced compounds sustaining denitrification.

### Nitrate turnover and its links to biogeochemical cycles in carbonate rock

The coexistence of microorganisms associated with nitrification, denitrification and anammox indicates that multiple nitrogen transformation pathways could operate simultaneously within the aquifer. Although nitrification is generally favoured under oxic conditions, we observed the potential for nitrification, aerobic ammonia oxidation, and aerobic nitrite oxidation along the aquifer transect (Table S6). Indeed, the *amoA* gene copy number was significantly higher in the recharge zone. In the anoxic zone, nitrogen cycle gene copies (*amoA, nirK*, and *nirS*) were significantly lower, with notable absences of *amoA* in all fractions sampled from site TBBrtz and in particle-associated fractions at sites TBAlt3 and TBEnt2 (Fig. 4).

The observed decline in the quantity of nitrogen cycle genes, as well as 16S rRNA bacterial and archaeal genes, towards the anoxic zone is likely indicative of an increase in energetic limitation caused by a depletion of both DOC and terminal electron acceptors (e.g. O_2_ and NO_3_⁻). Such reductions in microbial biomass are characteristic of oligotrophic groundwater systems with long residence times and restricted carbon availability.

While these qPCR-derived trends indicate clear spatial gradients across hydrogeological zones, they do not directly translate into microbial cell abundances or metabolic activity, particularly given potential differences in primer coverage and amplification efficiency between bacterial and archaeal assays. Therefore, the observed decreases are interpreted as qualitative indicators of reduced microbial potential under energy-limited conditions, rather than quantitative evidence of proportional biomass decline.

The inverse relationship between NO_3_⁻ concentrations and denitrification-associated metabolisms (nitrogen and nitrate respiration; Fig. 8) suggests that microbial NO_3_⁻ reduction could represent a major sink for NO_3_⁻ within the aquifer. In the recharge zone, nitrate reduction is likely supported primarily by heterotrophic respiration fueled by recharge-derived organic carbon, as indicated by its significant negative correlation with anaerobic chemoheterotrophy (Fig. 8). However, the persistence of NO_3_⁻ turnover under DOC-limited conditions in the anoxic zone indicates an increasing contribution of chemolithotrophic pathways linked to RSCs, Fe(II), and H_2_ oxidation.

Reduced sulfur compounds derived from pyrite-bearing carbonate rocks likely represent an important electron donor source for NO_3_⁻ reduction in the anoxic aquifer. The oxidation of pyrite can couple S^2^⁻ oxidation to denitrification, generating SO_4_^2^⁻. The relatively stable and elevated SO_4_^2^⁻ concentrations observed in the anoxic zone (105–186 mg/l) therefore support the hypothesis that RSCs-driven NO_3_⁻ reduction contributes to groundwater redox evolution.

The pyrite content ranged from 0.5%–1.0% in the tempestite facies and 0.5%–2.0% in the basinal facies of the Upper Muschelkalk. This content could provide a sustained, reduced inorganic substrate for microbial metabolism. Under oxic recharge conditions, pyrite oxidation is likely dominated by O_2_, whereas under O_2_-limited conditions, NO_3_⁻ may become the primary terminal electron acceptor, driving RSCs and Fe(II) oxidation.

Pyrite oxidation could additionally release Fe^2+^, thereby linking NO_3_⁻ turnover to Fe cycling within the aquifer. Although correlations between NO_3_⁻ depletion and iron oxidation were weaker than for sulfur oxidation, the occurrence of *Gallionella* and other Fe(II)-oxidizing taxa suggests that NRFeOx may contribute locally to denitrification processes. In bicarbonate-rich groundwater, dissolved Fe^2+^ may subsequently precipitate as siderite, which could potentially limit dissolved Fe accumulation despite ongoing Fe cycling.

Hydrogen may represent an additional electron donor supporting NO_3_⁻ reduction in both recharge and anoxic groundwater. Alongside the abiotic theory of the origin of H_2_ in groundwater, fermentative microorganisms can degrade organic carbon and release dissolved organic molecules (e.g. acetate) and H_2_. Microorganisms potentially capable of coupling H_2_ oxidation with NO_3_⁻ reduction were found at all sites (Fig. 3), with TBEnt2 (anoxic zone) showing the highest relative abundance of known H_2_ oxidizers. The widespread occurrence of microorganisms capable of hydrogen oxidation, together with predicted fermentation potential across the aquifer, may indicate active coupling between fermentative carbon degradation and hydrogenotrophic denitrification.

Acetate and H_2_ could support acetoclastic and hydrogenotrophic methanogenesis, with hydrogenotrophic methanogens detected in the recharge zone. Although dissolved CH_4_ was not detected during sampling, the presence of methanogenic and methanotrophic taxa suggests that localized CH_4_ production and oxidation could occur within anoxic microenvironments or biofilms. The potential for methanotrophic and methylotrophic activity was also observed throughout the aquifer. Thus, the occurrence of *Candidatus* Methylomirabilis in Has4 and in TBAlt3 and TBPol2 indicates the potential for NO^2^⁻-dependent anaerobic C1 compound oxidation (Yao et al. 2024).

Overall, the NO_3_⁻ turnover in the carbonate aquifer may be coupled to the oxidation of organic carbon, RSCs, Fe(II), H_2_, and potentially CH_4_. Within the recharge zone, the process of NO_3_⁻-reduction appears to be predominantly influenced by heterotrophic microorganisms, which are sustained by organic carbon derived from the recharge. In contrast, the anoxic zone is likely to rely increasingly on chemolithotrophic denitrification facilitated by reduced inorganic compounds.

### Hypothetical model of NO_3_⁻ turnover at the karstified and fractured aquifer

Nitrate turnover in the karstified and fractured carbonate aquifer is controlled by the availability of electron donors, electron acceptors, and hydrological transport processes. Recharge introduces O_2_, NO_3_⁻, DOC, and microorganisms from surface environments into the aquifer, thereby stimulating microbial activity within oxic and microoxic groundwater zones.

**Figure 9:**
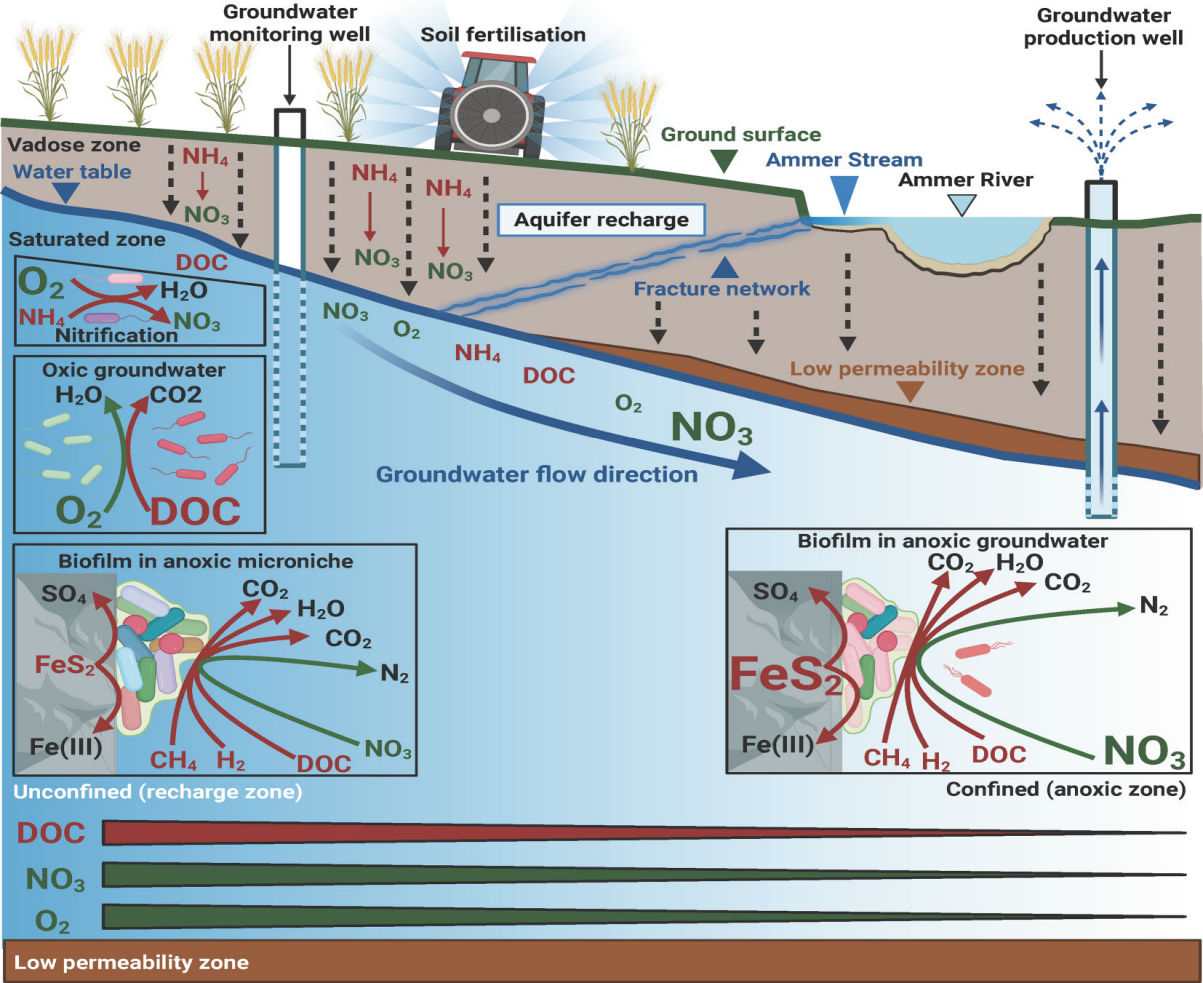
Hypothetical model of denitrification in the carbonate aquifer of the Ammer River catchment. The diagram illustrates the infiltration of NO_3_⁻ from soil fertilizer, DOC, and PO_4_^3^⁻ into groundwater within the recharge zone (unconfined oxic zone) and its subsequent transport to the anoxic zones (confined zone). Nitrifying microorganisms could further replenish the aquifer with NO_3_⁻ by oxidizing NH_4_^+^ in the recharge zone of the aquifer. Under the oxic conditions of the recharge zone, NO_3_⁻ may be reduced to N_2_ within anoxic microniches (e.g. within biofilms). In this process, the reduction of NO_3_⁻ could be coupled with the oxidation of CH_4_, H_2_, FeS_2_, and primarily bioavailable DOC. In the anoxic zone, particle- or biofilm-associated microorganisms could use NO_3_⁻ as a terminal electron acceptor, promoting the oxidation of CH_4_, H_2_, residual bioavailable DOC, and primarily FeS_2_. The activity of groundwater microbial communities leads to a decrease in DOC, O_2_, and NO_3_⁻ concentrations in the anoxic zone. Created in BioRender. Abramov, S. (2026) https://BioRender.com/wgfa1jp.

In the recharge zone, elevated DOC concentrations likely favour aerobic and facultatively anaerobic heterotrophic microorganisms capable of degrading organic matter. Aerobic respiration and nitrification probably act together to deplete the dissolved O_2_ in recharge groundwater. This depletion promotes the formation of localized microoxic or anoxic niches and could regenerate internal NO_3_⁻ within the recharge zone (Wegner et al. 2019). In these local niches, taxa closely related to anaerobic heterotrophs (e.g. *Brevundimonas, Polaromonas*, and *Cutibacterium*) and autotrophs (e.g. *Ralstonia*) could become active (Sorokina et al. 2012) (Fig. 9). However, as groundwater flows along the aquifer and DOC becomes depleted, alternative electron donors may increasingly sustain denitrification.

Reduced sulfur compounds and Fe(II) derived from pyrite could support autotrophic or mixotrophic NO_3_⁻ reduction. Taxa closely related to *Aquabacterium* and *Gallionella* may be involved in NO_3_⁻ reduction coupled with the oxidation of pyrite or siderite under autotrophic conditions (Fig. 8). While RSCs could modulate NO_3_⁻-reduction by *Rhodoferax, Sediminibacterium*, and *Sulfurifustis* (Taubert et al. 2019).

Hydrogen may additionally support NO_3_⁻ reduction, originating from both the fermentation of organic matter and water-rock interactions. The widespread detection of H_2_-oxidizing microorganisms suggests that hydrogenotrophic denitrification could be an important process under oligotrophic conditions.

Although methanogens were present at relatively low abundance, CH_4_ produced within biofilms or sediment-associated microenvironments could subsequently fuel aerobic (*Methylobacterium*; previously *Methylorubrum*) or anaerobic (*Candidatus* Methylomirabilis) CH_4_ oxidation. The occurrence of *Candidatus* Methylomirabilis suggests the potential for NO_2_^−^-dependent anaerobic CH_4_ oxidation, thereby linking CH_4_ turnover directly to denitrification processes.

The anoxic zone of the aquifer was characterized by oligotrophic conditions, including low DOC, O_2_, NO_3_⁻, and PO_4_^3^⁻ concentrations. The overall reduction in bacterial and archaeal 16S rRNA gene numbers likely reflects increasing energetic limitation along groundwater flow paths. Nevertheless, the persistence of heterotrophic and facultatively anaerobic taxa throughout the aquifer indicates substantial physiological adaptation to carbon- and energy-limited groundwater environments.

The proposed model describes a transition from heterotrophic NO_3_⁻ reduction in recharge groundwater, supported by surface-derived organic carbon. As groundwater becomes increasingly oligotrophic along the flow path, alternative inorganic electron donors likely sustain continued denitrification activity. This conceptual model highlights the importance of biogeochemical interactions for sustaining NO_3_⁻ turnover in carbonate aquifers under energy-limited conditions.

## Conclusions

Differences in O_2_, DOC, and NO_3_⁻ concentrations between the recharge and anoxic zones were associated with distinct microbial community compositions and predicted functional profiles in the carbonate aquifer. Microbial communities also varied among particle-size fractions and sampling locations, indicating the influence of hydrochemical conditions, recharge-derived inputs, and local aquifer characteristics.

The recharge zone was characterized by higher DOC and O₂ concentrations and a greater relative abundance of taxa associated with aerobic and facultatively anaerobic metabolisms. In contrast, the anoxic zone contained lower DOC concentrations and a microbial community enriched in taxa previously linked to denitrification and the oxidation of reduced inorganic compounds. The occurrence of microorganisms associated with Fe(II) and RSCs oxidation, together with elevated SO_4_^2−^ concentrations and the presence of pyrite-bearing carbonate rocks, suggests that lithology-derived electron donors could contribute to NO₃⁻ turnover under anoxic conditions.

Thus, the results indicate that hydrochemical gradients across the aquifer are closely linked to shifts in microbial community composition and potential nitrogen-cycling processes. The observed patterns are consistent with a transition from predominantly organic carbon-supported NO_3_⁻ turnover in recharge groundwater towards an increasing importance of alternative electron donors under DOC-limited conditions in the anoxic zone.

## Supplementary Material

fiag065_Supplemental_Files
