# Supplementary material for: Versatile microbial community responsible for nitrate turnover in a carbonate aquifer in southwest Germany
Source: FEMS Microbiol Ecol. 2026 Jun 30;102(8):fiag065. doi: 10.1093/femsec/fiag065 (PMC13377637; doi:10.1093/femsec/fiag065)
Supplement: fiag065_Supplemental_Files [file fiag065_supplemental_files.zip › Abramov-Manuscript-SI-FEMS.docx]

**Supplementary Materials for**

**Versatile microbial community responsible for nitrate turnover in a carbonate aquifer in southwest Germany**

Sergey Abramov^1,2#^, Nia Blackwell^1^, Karsten Osenbrück^1,3^, Daniel Straub^1,4^, Sven Nahnsen^4,5^, Andreas Kappler^1,6,7^, Peter Grathwohl^1^, Sara Kleindienst^1,2#^

^1^Department of Geoscience, University of Tuebingen, 72076 Tuebingen, Germany

^2^Institute of Sanitary Engineering, Water Quality and Solid Waste Management, University of Stuttgart, 70569 Stuttgart, Germany

^3^Federal Institute for Geosciences and Natural Resources (BGR), 30655 Hannover, Germany

^4^Quantitative Biology Center (QBiC), University of Tuebingen, 72076 Tuebingen, Germany

^5^Institute for Bioinformatics and Medical Informatics, University of Tuebingen, 72076 Tuebingen, Germany

^6^Cluster of Excellence: EXC 2124, Controlling Microbes to Fight Infection, University of Tuebingen, Tuebingen, 72076 Tuebingen, Germany

^7^Cluster of Excellence: EXC 3121, TERRA – Terrestrial Geo-Biosphere Interactions in a Changing World, University of Tübingen, 72076 Tuebingen, Germany

^#^Address correspondence to

Sergey Abramov: Department of Environmental Microbiology, University of Stuttgart, Institute for Sanitary Engineering, Water Quality and Solid Waste Management (ISWA)

Bandtäle 2, 70569 Stuttgart, Germany

Tel.: +49-(0)711-685-65852

Email: [sergey.abramov@iswa.uni-stuttgart.de](mailto:sergey.abramov@iswa.uni-stuttgart.de)

Sara Kleindienst: Department of Environmental Microbiology, University of Stuttgart, Institute for Sanitary Engineering, Water Quality and Solid Waste Management (ISWA)

Bandtäle 2, 70569 Stuttgart, Germany

Tel.: +49-(0)711-685-69351

Email: [sara.kleindienst@iswa.uni-stuttgart.de](mailto:sara.kleindienst@iswa.uni-stuttgart.de)

For submission to FEMS Microbiology Ecology

**Table S1.** Geological characteristics of the i) monitoring wells (mw): Sul 1, Sul3, Sul4, Moz1, Moz3, Moz4, Has3a, Has4, Has5; ii) karstic spring (ks): AMQ; and iii) production wells (pw) TBAlt3, TBPol2, TBEnt2 and TBBrtz. Geology symbols: mo (Upper Muschelkalk), mo1 (Unterer Hauptmuschelkalk), mo2 (Oberer Hauptmuschelkalk), mo2DO (Trigonodusdolomit), mo1HH (Haßmersheim-Schichten), mmDo (Oberer Dolomit), ku (Lower Keuper), q (Quartär)***.***

| **Field site** | **Sampling date** | **Depth**  **(m bgs)** | **Sampling depth**  **(m bwt)** | **Filter screen**  **(m bgs)** | | **Attitude (m asl)** | **Longitude** | **Latitude** | **Type** | **Aquifer geology** | **Cover geology (extent in m)** | **NH_4_^+^ (mg/l)** | **CH_4_ (ppm)** |  |
| --- | --- | --- | --- | --- | --- | --- | --- | --- | --- | --- | --- | --- | --- | --- |
|  |  |  |  |  |  |  |  |  |  |  |  |  |  |  |
| Sul4 | 17.01.18 | 42.5 | 39 | 32.0 | 42.0 | 547.1 | 8.7943 | 48.622 | mw | mo1/mmDo | q (1), mo (31) | <0.1 | <0.5 |  |
| Sul3 | 16.01.18 | 36.5 | 33 | 21.0 | 31.0 | 510.9 | 8.7952 | 48.6156 | mw | mmDo | mo (19) | <0.1 | NA |  |
| Sul1 | 16.01.18 | 92.0 | 83.0 | 68.0 | 88.0 | 576.0 | 8.8007 | 48.6221 | mw | mo1 / mmDo | mo (68) | <0.1 | <0.5 |  |
| Moz4 | 29.01.18 | 99.0 | 94.0 | 82.0 | 99.0 | 545.7 | 8.7679 | 48.5395 | mw | mo1/mmDo | q (1), ku (3), mo (81) | <0.1 | <0.5 |  |
| Moz3 | 29.01.18 | 111.0 | 98.0 | 92.0 | 102.0 | 553.5 | 8.7679 | 48.5463 | mw | mmDo | q (1), ku (6), mo (83) | <0.1 | <0.5 |  |
| Moz1 | 22.01.18 | 70.0 | 58 | 55.0 | 70.0 | 519.2 | 8.7619 | 48.5416 | mw | mo1/mmDo | mo2 (18), mo1/HH (37) | <0.1 | NA |  |
| Has5 | 23.01.18 | 87.7 | 82 | 82.0 | 87.0 | 490.7 | 8.8372 | 48.59 | mw | mo1/mmDo | q (1), mo (81) | <0.1 | <0.5 |  |
| Has4 | 24.01.18 | 80.0 | 70 | 70.0 | 80.0 | 469.3 | 8.8436 | 48.5884 | mw | mo1/mo1HH | q (2), ku (15), mo (53) | <0.1 | NA |  |
| Has3a | 23.01.18 | 58.0 | 54.0 | 50.0 | 58.0 | 553.5 | 8.7679 | 48.5463 | mw | mmDo | q (1), ku (6), mo (83) | <0.1 | <0.5 |  |
| AMQ | 04.01.18 | NA | NA | NA | NA | 403 | 8.8554 | 48.5843 | ks | mo2 | na | <0.1 | NA |  |
| TBAlt3 | 31.01.18 | 25.5 | 58.5 | 25.5 | 58.5 | 377 | 8.9129 | 48.5549 | pw | mo2/mo1 | q (6), ku (10), mo2Do (35) | <0.1 | NA |  |
| TBBrtz | 06.02.18 | 65.0 | 65 | 41.0 | 62.2 | 395.7 | 8.9503 | 48.5657 | pw | mo2/mo1 | na | <0.1 | <0.5 |  |
| TBEnt2 | 30.01.18 | 43.3 | 43.3 | 25.0 | 38.0 | 365 | 8.9629 | 48.5397 | pw | mo2/mo1 | q (7m), ku (9m), mo2Do (13m) | <0.1 | <0.5 |  |
| TBPol2 | 07.02.18 | 45.6 | 45.6 | 10.6 | 38.6 | 350 | 8.9573 | 48.5314 | pw | mo2 | q (7m), ku (2m), mo2Do (16m) | <0.1 | <0.5 |  |

**Table S2 (see Table S2.xlsx).** Number of PCR and booster PCR cycles used to amplify DNA extracted from groundwater samples.

**Table S3**. Primers and protocols used to quantify functional genes encoding ammonia monooxygenase (*amoA*), nitrite reductases (*nirK* and *nirS*) and the bacterial and archaeal 16S rRNA genes.

| **Gene** | **Primers** | **bp number** | **Reference** | **PCR protocol** |
| --- | --- | --- | --- | --- |
| *nirK* | nirK876c ATYGGCGGVCAYGGCGA  nirK1040 GCCTCGATCAGRTTRTGGTT (modified) | 164 | [10.1128/AEM.02013-17](https://doi.org/10.1128/AEM.02013-17) | 98°C - 10 s, 58°C - 20 s  × 40 cycles |
| *nirS* | nirSCd3aF AACGYSAAGGARACSGG  nirSRcd GASTTCGGRTGSGTCTTSAYGAA | 407 | [10.1128/AEM.02013-17](https://doi.org/10.1128/AEM.02013-17) | 98°C - 30 s, 57°C - 30 s, 72°C - 30 s  × 40 cycles |
| 16S rRNA gene | 341f CCTACGGGAGGCAGCAG  797r GGACTACCAGGGTATCTAATCCTGTT | 456 | [10.1128/AEM.02013-17](https://doi.org/10.1128/AEM.02013-17) | 98°C - 5 s,  60°C - 12 s, 95°C - 1 min  × 40 cycles |
| 16S rRNA genes of  *Archaea* | Ar109f  ACK GCT GAG TAA CAC GT  Ar915r  GTG CTC CCC CGC CAA TTC CT | 800 | <https://doi.org/10.1016/j.envpol.2021.117012> | 98°C - 3 min,  (98°C - 5 s, 52°C - 12 s,  72°C - 15 s)  × 40 cycles,  98°C - 1 min, 52°C – 1 min, 52 - 95°C – 10 min |
| archaeal  *amoA* | amo19F ATGGTCTGGCTWAGACG  CrenamoA616r48x GCCATCCABCKRTANGTCCA | 624 | https://doi.org/10.1038/ismej.2013.160 | 98°C - 45 s  55°C - 45 s  72°C - 45 s  × 40 cycles |

**Table S4 (see Table S4.xlsx).** Pairwise post-hoc comparisons of Shannon indexes.

**Table S5 (see Table S5.xlsx).** Functional predictions (selected functions) of particle-associated (>8 µm) and planktonic (0.4-8 µm and 0.2-04 µm) fractions of groundwater microbial communities sampled from field sites located along the Ammer River catchment (SW Germany). Functional predictions were performed using FAPROTAX v1.2.10 (Louca et al. 2016). Generated in RStudio Version 2024.12.1+563 (R version 4.4.2) with the microeco package applied.

**
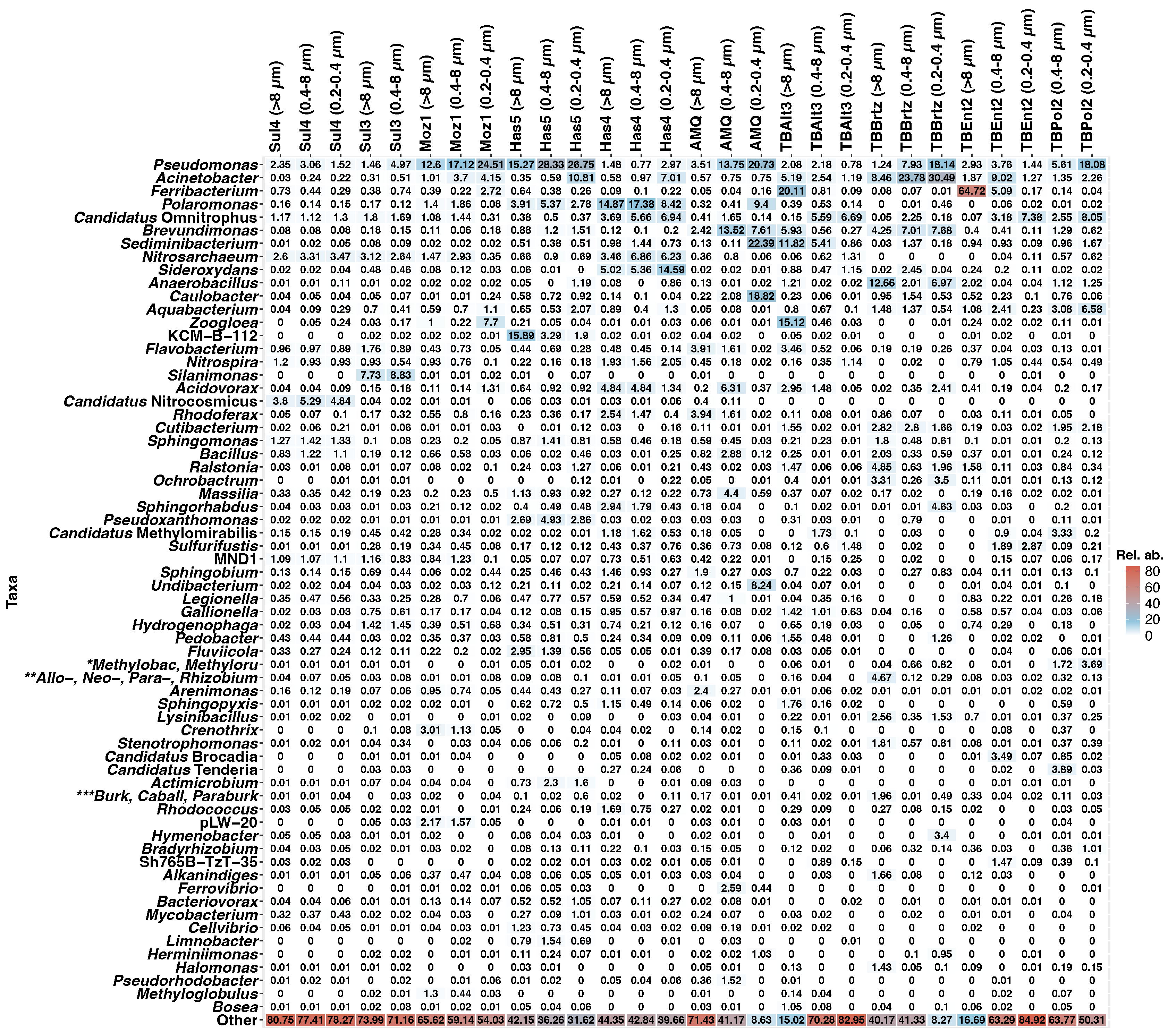
**

**Fig. S1.** Relative abundance of 65 microbial genera identified in particle-associated (>8 µm) and planktonic (0.4-8 µm and 0.2-0.4 µm) fractions of groundwater microbial communities sampled from field sites located along the Ammer River catchment (SW-Germany). The figure shows only those genera with >1% of relative abundance in at least one sample. **Methylobac*, *Methylorub* is an acronym for *Methylobacterium*-*Methylorubrum*. ***Allo-*, *Neo-*, *Para-*, *Rhizobium* is an acronym for *Allorhizobium-Neorhizobium-Pararhizobium-Rhizobium*. ****Burk*, *Caball*, *Paraburk* is an acronym for *Burkholderia*-*Caballeronia*-*Paraburkholderia.* Generated in RStudio Version 2024.12.1+563 (R version 4.4.2) with the microeco package applied.


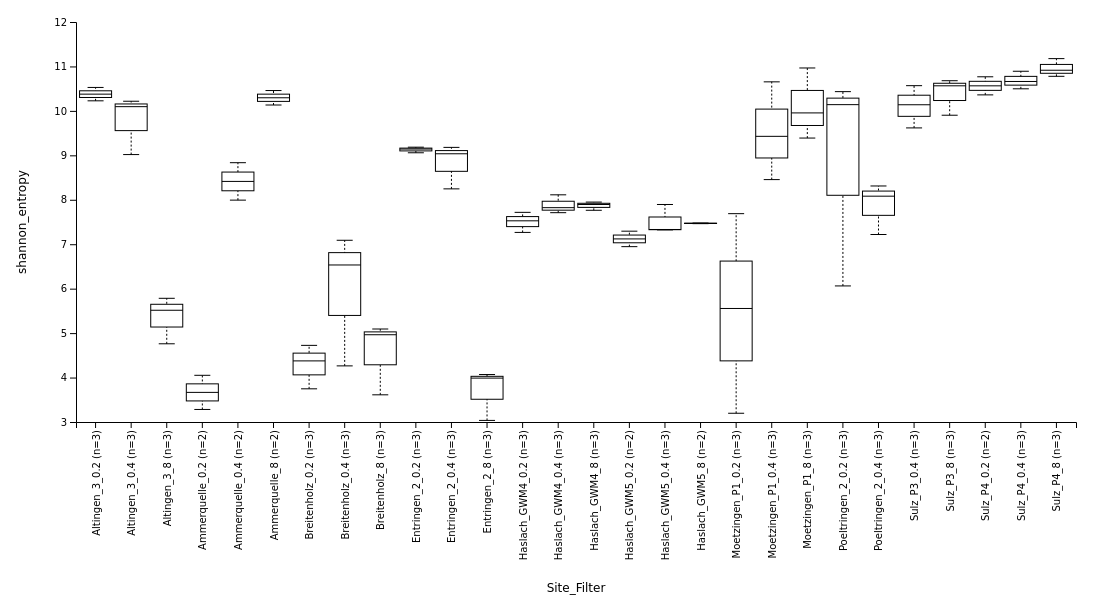


**Fig S2.** Shannon diversity of groundwater microbial communities from i) monitoring wells: Sul3, Sul4, Moz1, Has4, and Has5; ii) karstic spring: AMQ; and iii) production wells: TBAlt3, TBPol2, TBEnt2, and TBBrtz.

**Fig. S3.** Non-metric multidimensional scaling (NMDS) visualizing overall patterns in microbial community composition independent of environmental constraints.

**Fig. S4.** Analysis of multivariate dispersion based on Bray-Curtis dissimilarities among sampling sites.

**Fig. S5.** Random Forest (rf) analysis showing the importance of microbial genera in distinguishing groundwater microbial communities across sampling sites (Sul4, Sul3, Moz1, Has5, Has4, AMQ, TBAlt3, TBBrtz, TBEnt2, and TBPol2). Taxonomic importance was evaluated using MeanDecreaseGini scores, where higher values indicate a greater contribution of a genus to site classification. The identified taxa therefore represent discriminatory features associated with sites, rather than statistically independent site-specific biomarkers. To identify discriminatory taxa, microbial communities replicate samples originating from the same site and size fraction were analysed. Generated in RStudio Version 2024.12.1+563 (R version 4.4.2) using the microeco package.

**Fig S6.** Comparison of 16S rRNA bacterial and archaeal, *nirK*, *nirS* and *amoA* gene copy numbers across sampling zones, and comparison of 16S rRNA bacterial and archaeal gene copy numbers across all samples. **A.** Bacterial 16S rRNA gene copy numbers across zones. Boxplots show median values (Recharge: 1,140,736.6, Anoxic: 11,723.2) with interquartile ranges (boxes), whiskers extending to 1.5 × IQR, and individual points representing site averages (n=12 Recharge, 11 Anoxic sites). The difference across zones was significant (Wilcoxon rank-sum test, W=103, 0.01<p≤0.05). **B.** Archaeal 16S rRNA gene copy numbers across zones. Boxplots show median values (Recharge: 544,755.5, Anoxic: 230.4) with interquartile ranges (boxes), whiskers extending to 1.5×IQR, and individual points representing site averages (n=12 Recharge, 11 Anoxic sites). The difference across zones was significant (Wilcoxon rank-sum test, W=118, p≤0.001). **C.** Comparison of 16S rRNA gene copy numbers between Archaeal and Bacterial domains. Boxplots show median values (Archaeal: 25,354.9, Bacterial: 312,511.2) with interquartile ranges (boxes), whiskers extending to 1.5 × IQR, and individual points representing site averages (n=23 Archaeal, 23 Bacterial sites). The difference between domains was significant (Wilcoxon rank-sum test, W=123.5, 0.01<p≤0.05). **D.** *nirK* gene copy numbers across zones. Boxplots show median values (Recharge: 108,000.91, Anoxic: 497.05) with interquartile ranges (boxes), whiskers extending to 1.5×IQR, and individual points representing site averages (n=12 Recharge, 10 Anoxic sites). The difference across zones was significant (Wilcoxon rank-sum test, W=115, p≤0.001). **E.** *nirS* gene copy numbers across zones. Boxplots show median values (Recharge: 862,787.55, Anoxic: 311.18) with interquartile ranges (boxes), whiskers extending to 1.5 × IQR, and individual points representing site averages (n=12 Recharge, 11 Anoxic sites). The difference across zones was significant (Wilcoxon rank-sum test, W=122, p≤0.001). **F.** *amoA* gene copy numbers across zones. Boxplots show median values (Recharge: 219,103.96, Anoxic: 57.68) with interquartile ranges (boxes), whiskers extending to 1.5×IQR, and individual points representing site averages (n=12 Recharge, 9 Anoxic sites). The difference across zones was significant (Wilcoxon rank-sum test, W=95, p≤0.01). All analyses were performed in R version 4.4.2 using non-parametric Wilcoxon rank-sum tests with continuity correction (due to non-normal distribution of gene copy numbers). Boxplot elements represent center line = median, box limits = 25th-75th percentiles, whiskers = 1.5 × IQR. Analyses used dplyr for data processing, ggplot2 for visualization, and ggpubr for statistical annotations. All gene copy numbers were log10-transformed for visualization while maintaining the original scale for statistical tests.

**Fig. S7.** Comparison of microbial functional potentials across sampling zones based on FAPROTAX analysis (Louca et al. 2016; Sansupa et al. 2021). **A.** Chemoheterotrophy potential across zones. Boxplots show median values (Recharge: 29.7, Anoxic: 29.6) with interquartile ranges (boxes), whiskers extending to 1.5 × IQR, and individual points representing site averages (n=18 Recharge, 11 Anoxic sites). The difference across zones was not significant (Wilcoxon rank-sum test, W=103, p>0.05). **B.** Aerobic chemoheterotrophy potential across zones. Boxplots show median values (Recharge: 23.8, Anoxic: 22.2) with interquartile ranges (boxes), whiskers extending to 1.5 × IQR, and individual points representing site averages (n=18 Recharge, 11 Anoxic sites). The difference across zones was not significant (Wilcoxon rank-sum test, W=109, p>0.05). **C.** Anaerobic chemoheterotrophy potential across zones. Boxplots show median values (Recharge: 5.6, Anoxic: 6.6) with interquartile ranges (boxes), whiskers extending to 1.5 × IQR, and individual points representing site averages (n=18 Recharge, 11 Anoxic sites). The difference across zones was not significant (Wilcoxon rank-sum test, W=58, p>0.05). **D.** Methanotrophy potential across zones. Boxplots show median values (Recharge: 1.048, Anoxic: 1.113) with interquartile ranges (boxes), whiskers extending to 1.5 × IQR, and individual points representing site averages (n=18 Recharge, 11 Anoxic sites). The difference across zones was not significant (Wilcoxon rank-sum test, W=98, p>0.05). **E.** Methylotrophy potential across zones. Boxplots show median values (Recharge: 1.876, Anoxic: 1.463) with interquartile ranges (boxes), whiskers extending to 1.5 × IQR, and individual points representing site averages (n=18 Recharge, 11 Anoxic sites). The difference across zones was not significant (Wilcoxon rank-sum test, W=121, p>0.05). **F.** Dark (non-photosynthetic) oxidation of sulfur compounds across zones. Boxplots show median values (Recharge: 0.382, Anoxic: 0.523) with interquartile ranges (boxes), whiskers extending to 1.5 × IQR, and individual points representing site averages (n=18 Recharge, 11 Anoxic sites). The difference across zones was significant (Wilcoxon rank-sum test, W=54.5, 0.01<p≤0.05). **G.** Dark (non-photosynthetic) sulfide oxidation across zones. Boxplots show median values (Recharge: 0.185, Anoxic: 0.297) with interquartile ranges (boxes), whiskers extending to 1.5 × IQR, and individual points representing site averages (n=18 Recharge, 11 Anoxic sites). The difference across zones was not significant (Wilcoxon rank-sum test, W=76.5, p>0.05). **H.** Dark (non-photosynthetic) iron oxidation across zones. Boxplots show median values (Recharge: 0.17, Anoxic: 0.37) with interquartile ranges (boxes), whiskers extending to 1.5×IQR, and individual points representing site averages (n=18 Recharge, 11 Anoxic sites). The difference across zones was significant (Wilcoxon rank-sum test, W=26.5, 0.001<p≤0.01). **I.** Iron respiration across zones. Boxplots show median values (Recharge: 0.465, Anoxic: 0.737) with interquartile ranges (boxes), whiskers extending to 1.5×IQR, and individual points representing site averages (n=18 Recharge, 11 Anoxic sites). The difference across zones was significant (Wilcoxon rank-sum test, W=18, p≤0.001). **J.** Dark (non-photosynthetic) hydrogen oxidation across zones. Boxplots show median values (Recharge: 1.587, Anoxic: 1.737) with interquartile ranges (boxes), whiskers extending to 1.5 × IQR, and individual points representing site averages (n=18 Recharge, 11 Anoxic sites). The difference across zones was not significant (Wilcoxon rank-sum test, W=76, p>0.05). All analyses were performed in R version 4.4.2 using non-parametric Wilcoxon rank-sum tests with continuity correction. Boxplot elements represent center line = median, box limits = 25th-75th percentiles, whiskers = 1.5 × IQR. Analyses used dplyr for data processing, ggplot2 for visualization, and rstatix for statistical annotations.
